# Geomorphology of the Mirador-Calakmul Karst Basin: A GIS-based approach to hydrogeologic mapping

**DOI:** 10.1371/journal.pone.0255496

**Published:** 2021-08-02

**Authors:** Ross Ensley, Richard D. Hansen, Carlos Morales-Aguilar, Josie Thompson

**Affiliations:** 1 Terra Geo Solutions, Houston, Texas, United States of America; 2 Department of Anthropology, University of Utah, Salt Lake City, Utah, United States of America; 3 Laboratoire Archéologie des Amériques UMR 8096-CNRS, Université Paris 1 Panthéon Sorbonne, Paris, France; 4 Mirador Conservation Fund, Redwood City, California, United States of America; University of Florida, UNITED STATES

## Abstract

This paper classifies the karst landscapes of the Petén Plateau and defines the Mirador-Calakmul Karst Basin by illustrating the distribution of its karst hydrologic features. Archaeological and spatial research of the Mirador-Calakmul area of Guatemala and Mexico has shown it to be a karst basin with geopolitical implications. Current research characterizes the karst landscapes of the Petén Plateau, maps the distribution of karst hydrologic features, and delineates the basin in geomorphological terms. To further this aim, multiple forms of remote sensing data including orthophotographs, a satellite Digital Elevation Model, satellite multispectral images, and Light Detection and Ranging (LiDAR) data have been integrated to interpret the karst features in the study area. Outcrop study and thin section analysis of the upper Buena Vista Formation document that the dominant lithologies are a shallow water algal boundstone interbedded with terrestrial caliche. Karst landforms have been mapped over the Petén Plateau and we identify five karst landscapes, the largest of which is a fluviokarst landscape dominated by karst valleys. We further map karst hydrologic features including seasonal swamps, dolines, intermittent lakes, intermittent streams, solution-enhanced fractures, and springs all of which are characteristic of drainage basins. Boundaries of the karst basin are mapped from multiple lines of evidence including distribution of the karst valleys, a line of springs along the western boundary of the fluviokarst landscape, and a surface drainage analysis. We capture and classify hydrologic data points and develop a regional groundwater map that indicates subsurface flow from east to west within the basin. A drainage map illustrates the extensive system of karst valleys, boundaries, and inferred groundwater flow paths of the Mirador-Calakmul Karst Basin. It was within this geomorphological setting that the ancient Maya developed an extensive civilization during the Middle and Late Preclassic periods (1000 BCE-150 CE).

## Introduction

The Mirador-Calakmul Karst Basin is a unique hydrogeological setting in the southern Yucatán Peninsula that fostered development of the incipient Maya civilization. Two large conservation areas, the Maya Biosphere and Calakmul Biosphere reserves lie within northern Petén, Guatemala, and southern Campeche, Mexico, respectively. These reserves were originally created to preserve the tropical forests and protect numerous ancient Maya settlements inside their borders. Multiple lines of research have documented the cultural significance of the region over the last four decades, but investigation of the geology has been sporadic. Our research seeks to describe the geomorphology of the basin and document it with a karst drainage map.

The central Yucatán Peninsula of Mexico and Guatemala is an upland region of low hills recognized by cartographers since early colonial times. Heilprin first described this hilly region in geologic terms as an axial fold associated with uplift [1], while Wadell was the first author to consider the hilly backbone a plateau [2]. Recent authors have divided the Yucatán into physiographic provinces centered around the Petén Plateau, bounded on the east by a broad zone of normal faults, and dipping to a coastal plain on the west [3–5]. Outcropping on the plateau in the Petén are Lower Paleogene limestones and evaporites of the Buena Vista Formation, broadly correlative with the Icaiché Formation in Campeche, both heretofore considered deposited in a shallow to restricted marine setting [6,7]. Within this geological setting, the rolling hills of the plateau and the flanking lowlands the ancient Maya developed early civilization.

Extensive karst landscapes have developed on the Petén Plateau given the tropical environment and the presence at surface of extensive carbonates. Karst landscapes, assemblages of karst landforms, are often dominated by one landform related to their hydrogeologic setting including structure, lithology, and hydrology [8–11]. Although the karst geomorphology of the Yucatán Peninsula has been described by numerous authors [12–15], only occasionally has the analysis been applied in a manner that recognizes the unique setting of the Petén Plateau. In his study of the geomorphology of the Calakmul Biosphere Reserve, Gates mapped the fluviokarst landscape of the central plateau with its karst valleys, flanked on the west by karst lagoons, swamps, and residual hills [16]. Heraud-Pina mapped the polje karst landscape of the northern plateau with its large-scale closed depressions, along with a karst plain and residual hills to the west and north of the plateau [17]. To our knowledge, however, no paper has documented the karst landscapes of the southern Petén Plateau extending from southern State of Campeche, Mexico, into northern Department of Petén, Guatemala.

Multiple authors have reviewed different topics on Maya politics, cultural development, and ecology in the basin, indicating its singular nature and antiquity. During the Late Preclassic period (350 BCE– 150 CE), El Mirador appears to have become the main center of the region. Subsequently, in the Classic period, Calakmul became one of the powerful capitals of the Maya lowlands [18–21]. Consideration of cultural continuity and internal trade suggests the use of a single polity when describing Calakmul, El Mirador and their surrounding settlements [22,23]. Examination of codex-style ceramics of the Late Classic period (ca. 700 CE) indicate manufacture exclusively within the Mirador-Calakmul region [24–26]. Consideration of Maya trade routes have shown that the Mirador-Calakmul region dominated Maya trans-peninsular trade in the Yucatán, first under El Mirador in the Late Preclassic (ca. 350 BCE) and later during the Late Classic (ca. 600 CE) under Calakmul [21,27]. Given the importance of the region as a contiguous cultural area, the karst landscape of the Petén Plateau merits classification and the hydrogeologic framework of the Mirador-Calakmul Karst Basin warrants definition.

Drainage in the southern Petén Plateau is controlled, both on the surface and in the subsurface, by naturally occurring karst hydrologic features. A mapping unit commonly used to delimit such areas is a karst drainage basin, the total area of surface and subsurface drainage that contributes to a conduit system and its outlet spring or springs [11,28–31]. Three primary components define such basins: karst hydrologic features, groundwater flow routes, and their boundaries. The hydrological features of a karst drainage basin, as outlined by Ray, are wetlands or swamps, dolines, intermittent lakes, a lack of streams, subsurface conduits, and springs [29]. One natural outcome of a hydrogeologic study is a drainage map of a karst basin that documents the surface landforms and the inferred subsurface flow. The karst features which have been mapped regionally on the southern plateau are the seasonal swamps, locally known as *bajos*, which are prevalent throughout the system of karst valleys [32–37]. Several studies have modeled groundwater flow over different portions of the Yucatán [38–42] and one model for the entire peninsula [43], all of which have demonstrated groundwater bidirectional flow from the central anticlines of the Petén Plateau to the west and east. However, studies to date have not mapped the full suite of karst hydrologic features consistently over the plateau nor delimited boundaries of karst basins. Geographic Information System (GIS) technology offers a convenient approach to mapping the hydrologic features, modeling subsurface flow paths, and delimiting boundaries of karst basins [11,30,44–47].

This paper aims to characterize the geomorphology and karst landscapes of the southern Petén Plateau and to define the Mirador-Calakmul region in a hydrogeological framework. Focus on this region is driven by environmental and archaeological research which indicates the development of the Maya civilization especially during the Middle (1000–350 BCE) and Late Preclassic (350 BCE– 150 CE) periods was influenced by karst geomorphology with a strong relationship between human activities and natural processes represented by different karst landscapes [34,48–50]. This paper presents a synopsis of the development of the Petén Plateau and documents the bounding Buenavista Fault with seismic data. We present evidence of previously unrecognized subaerial sedimentation within the upper Buena Vista Formation. We extend the early work of Gates and Heraud-Pina with a first map of the karst landscapes of the southern Petén Plateau and show that a fluviokarst landscape, with its primary landform of karst valleys, covers much of the plateau [16,17]. Our regional groundwater map provides a detailed view of aquifer flow in northern Petén and southern Campeche, complementing a peninsula-wide model [43], and demonstrates groundwater flow from the central anticlines of the plateau to the west. We provide the first hydrogeologic description of the Mirador-Calakmul Karst Basin (MCKB) and document it with a drainage map. This finding is based on a detailed mapping of karst hydrologic features throughout the southern plateau accomplished through the integration of multiple forms of remote sensing data and a GIS-based approach to mapping hydrogeology.

## Regional setting

**Fig 1:**
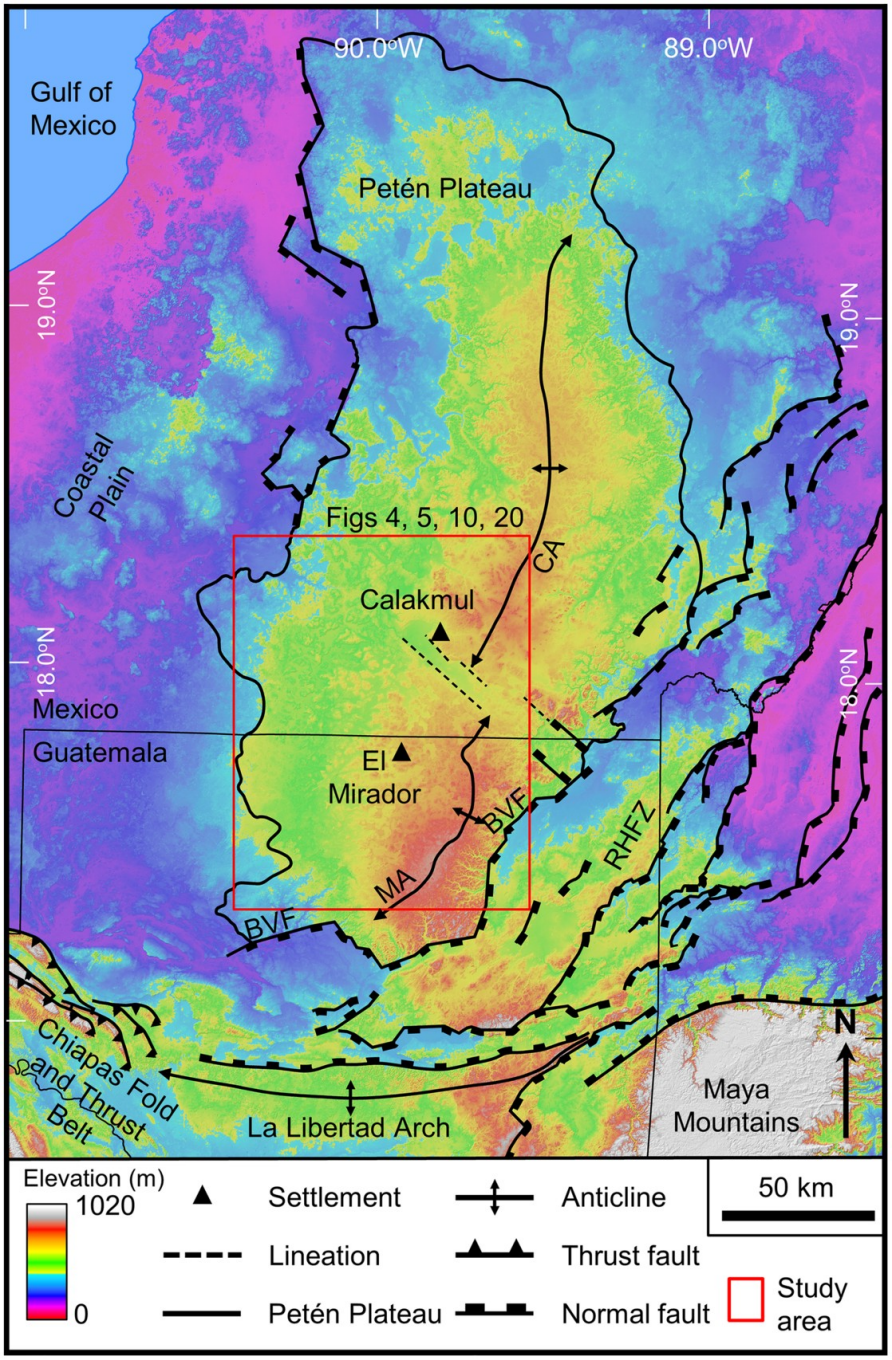
Physiographic map of the southern Yucatán Peninsula. The Yucatán Platform, bounded to the south along a line from the Chiapas Fold and Thrust Belt to the northern limits of the Maya Mountains, was a shallow marine depositional setting during the Paleocene-Eocene. Isostatic rebound during the middle Eocene uplifted the Petén Plateau (PP) and created the Mirador and Calakmul anticlines (MA and CA). Northwest striking lineations, in line with two short normal faults to the east, highlight a potential subsurface graben south of Calakmul. Pliocene extension down dropped the east side of the YP forming the Río Hondo Fault Zone (RHFZ) a structural province consisting of half grabens created by northeast trending normal faults. Key among these is the Buenavista Fault (BVF) that delimits the southeast margin of the plateau. ALOS World 3D (AW3D30) elevation data have been provided by the Japan Aerospace Exploration Agency (https://www.eorc.jaxa.jp/ALOS/en/aw3d30/) and printed under a CC BY 4.0 license.

The Yucatán Peninsula (YP) lies in southeast Mexico, northern Guatemala, and northern Belize and is bordered on the east by the Caribbean Sea, on the northwest by the Gulf of Mexico, and in the south by the La Libertad Arch [5]. Multiple authors have divided the peninsula into a few physiographic regions based on geologic structure and geomorphology [3–5,12], while others have subdivided the peninsula into a larger number of adaptive regions based on physiographic and environmental factors that have created a mosaic of habitats [51–53]. For a discussion of karst development, however, we subdivide the region into subregions that are each underpinned by a common structure style, geology, and surface geomorphology (Fig 1 and S1 File). Central among these is the Petén Plateau (PP), flanked by the Río Hondo Fault Zone (RHFZ) on the east and the coastal plain on the west.

The PP lies in the Petén Department of northern Guatemala and the Campeche State of southeastern Mexico [5]. It is bounded on the south, east, and northwest by normal faults that enclose an area of 26,930 km^2^ (S1 File). Along the south and southeastern sides of the plateau, the Buenavista Escarpment has a relief of up to 200 m [4,6,54] while faults on the northwest flank have a relief of only 30 m, providing a gentle overall west-northwest tilt [2,55]. The RHFZ, bordering the plateau to the east, first developed in Late Cretaceous to Early Paleocene as part of a left-lateral shear zone associated with the opening of the Yucatán Basin to the east, and formed a northeast trending forebulge in the Yucatán Platform [56–58]. Isostatic rebound of the YP occurred in the waning stages of the opening of the Yucatán Basin during Middle to Late Eocene (47.8 to 33.9 Ma) as the Yucatán Basin became welded to the North American Plate, uplifting Lower Paleogene carbonates [57–59]. Dates noted in this paper follow the Geologic Society of America Geologic Time Scale [60]. Subsequent reactivation of the RHFZ in an extensional regime during the Pliocene (5.3–2.6 Ma) formed the escarpments on the south and southeast margins of the plateau [37,60–62]. Other authors have also mapped the extent of the plateau similarly [63–66].

As a result of the uplift, the PP is dominated by an anticlinal structure [7,37,67,68], the northern end plunging gently to the north-northeast and the southern end plunging gently to the south-southwest. Although it has been discussed as a single structural feature, it is composed of two separate anticlines offset over 10 km by the El Laberinto Bajo [55], a karst valley (KV) that may overlie a Jurassic graben [69,70]. Recognizing the importance of these anticlines as the eastern border of the Mirador-Calakmul Karst Basin (MCKB), the authors refer to them as the Mirador Anticline (MA) in the south and the Calakmul Anticline (CA) in the north.

**Fig 2:**
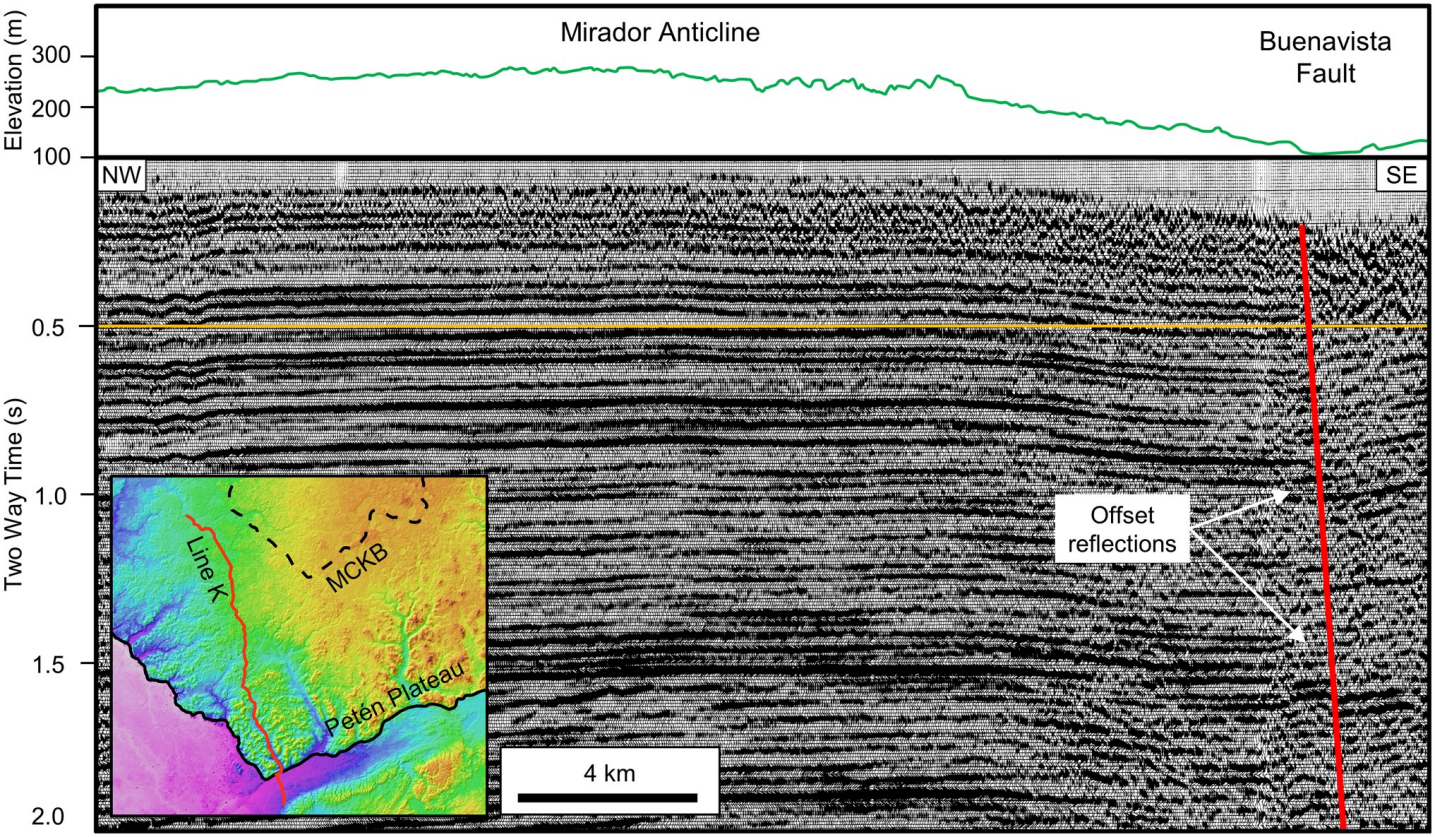
Seismic Line K over the southeastern Petén Plateau showing the Mirador Anticline and BVF. At the surface, the MA is a broad topographical high, while in the subsurface we interpret it based on a series of bowed reflections. Line K follows a dry valley down from the plateau and crosses the BVF at the village of San Miguel, where there is little surface expression or an escarpment. A few hundred meters southwest of this point, however, the escarpment is 150 m high. The orange timing line at 0.5 s highlights the gentle anticline. Seismic data are publicly available from the Guatemalan Ministero de Energía y Minas (https://mem.gob.gt/). AW3D30 elevation data have been provided by JAXA (https://www.eorc.jaxa.jp/ALOS/en/aw3d30/) and printed under a CC BY 4.0 license.

The present-day MCKB, developed within the Paleogene carbonates of the Buena Vista Formation, lies near the northern edge of the Mesozoic-Cenozoic North Petén sedimentary basin. As mapped by Dengo, the Petén Basin of Guatemala covers a region of 110,000 km^2^ and is bounded by the Chiapas massif to the west, the Polochic Fault to the South, the Maya Mountains to the east, and thins to the north over the Yucatán Platform [71]. The basin initially developed in the Late Jurassic as a rift basin evolving during the Early Cretaceous into a shallow marine basin. All total, the basin has over 12,000 m of sediment fill including the Jurassic red beds overlain by Cretaceous-Paleogene evaporites and carbonates. The La Libertad Arch separates the basin into the North Petén and South Petén sub-basins. More thorough descriptions of the structural history of northern Guatemala, the formation of the Petén Basin, and its stratigraphic fill have previously been published [6,67,71–73]. Exposed over much of the Guatemalan portion of the PP is the Buena Vista Formation, loosely correlative with the Icaiché Formation to the north. Vinson described the type section from the Buenavista Escarpment (Fig 2) along the southern edge of the plateau as containing Lower Eocene carbonates and evaporites [6]. Subsequently, Lopez Ramos assigned an age of Paleocene to Early Eocene, 66.0–47.8 Ma, to the Icaiché Formation [74], later confirmed by others [7,75,76].

**Fig 3:**
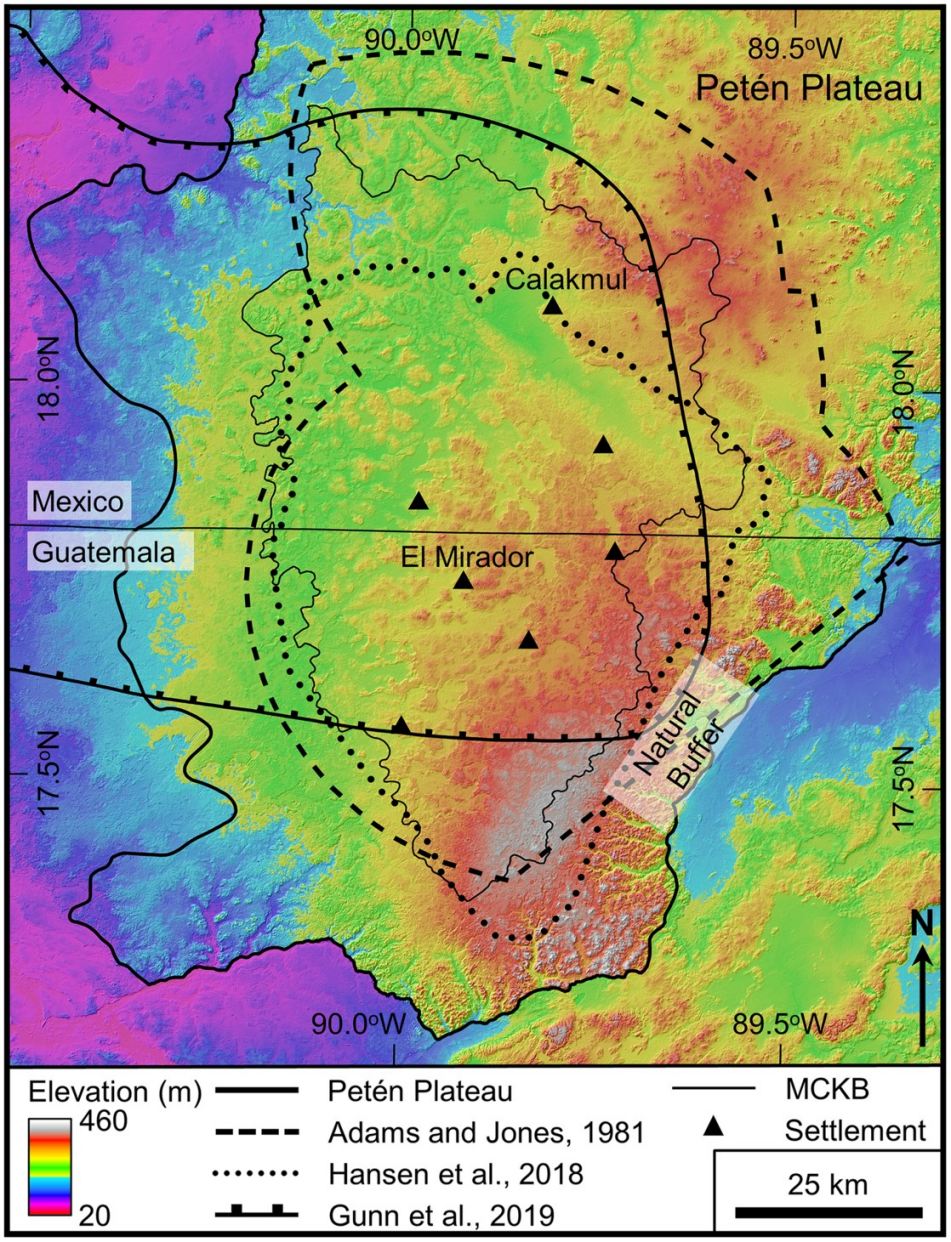
Map illustrating delineation of the Mirador-Calakmul region by earlier authors. Shown are the Calakmul Geographical Region [77], the upper Candelaria River Watershed referred to as the Calakmul Basin [21], and the Mirador-Calakmul Basin [85]. These compare closely to the boundary of the MCKB defined below. Also noted is a natural buffer zone, the polygonal karst landscape, a region which Gunn and others defined as a military buffer between the Tikal and Calakmul regions during the Classic period [21]. AW3D30 elevation data have been provided by JAXA (https://www.eorc.jaxa.jp/ALOS/en/aw3d30/) and printed under a CC BY 4.0 license.

There has been interest in the geomorphological nature of the Mirador-Calakmul area since it was first defined as a unique geographical region (Fig 3) as determined by natural boundaries, spatial contiguity, and architectural style [77–79]. Early authors informally referred to the Petén and Campeche portions of the region as the Mirador basin and the Calakmul basin, respectively, indicating that they were geographically separated from the rest of Petén by karstic hills surrounding the region [80–83]. Later, these same authors recognized how the two were part of a singular geographical system. Folan and others mapped the combined area as a riverine district, a description that emphasized the nature of the surface drainage basin [84], while Hansen and others [34,50] described the unified area based on its karst hydrologic features (seasonal swamps, dolines, intermittent ponds, and lack of perennial streams) in a manner that emphasized the subsurface drainage of the basin. To highlight the geomorphological and cultural continuity between northern Petén and southern Campeche, the name Mirador-Calakmul Basin has been used to describe the full area circumscribed by karstic hills on the north, east, south, and to a lesser degree on the west [34,85]. Authors mapped the borders of the basin based on geologic structure, surface drainage, and karst landforms [34,37,48,49]. This view of a unified cultural and geomorphological basin was echoed more recently by Folan, Gunn and other authors who mapped Mirador and Calakmul within a single watershed, emphasized the spatial contiguity of the El Mirador and Calakmul sites, and highlighted the natural buffer zone separating them from Tikal [21,86]. Since 1992 over 200 publications have referred to the region as a basin, and several authors have alluded to the karst influence on the hydrogeology. More recently, though, Ensley first described the Mirador-Calakmul region as a karst drainage basin [37]. It is worth noting that the Calakmul geographical region (Fig 3) originally defined by Adams and Jones closely resembles our contemporary view of the MCKB [77].

## Materials and methods

**Fig 4:**
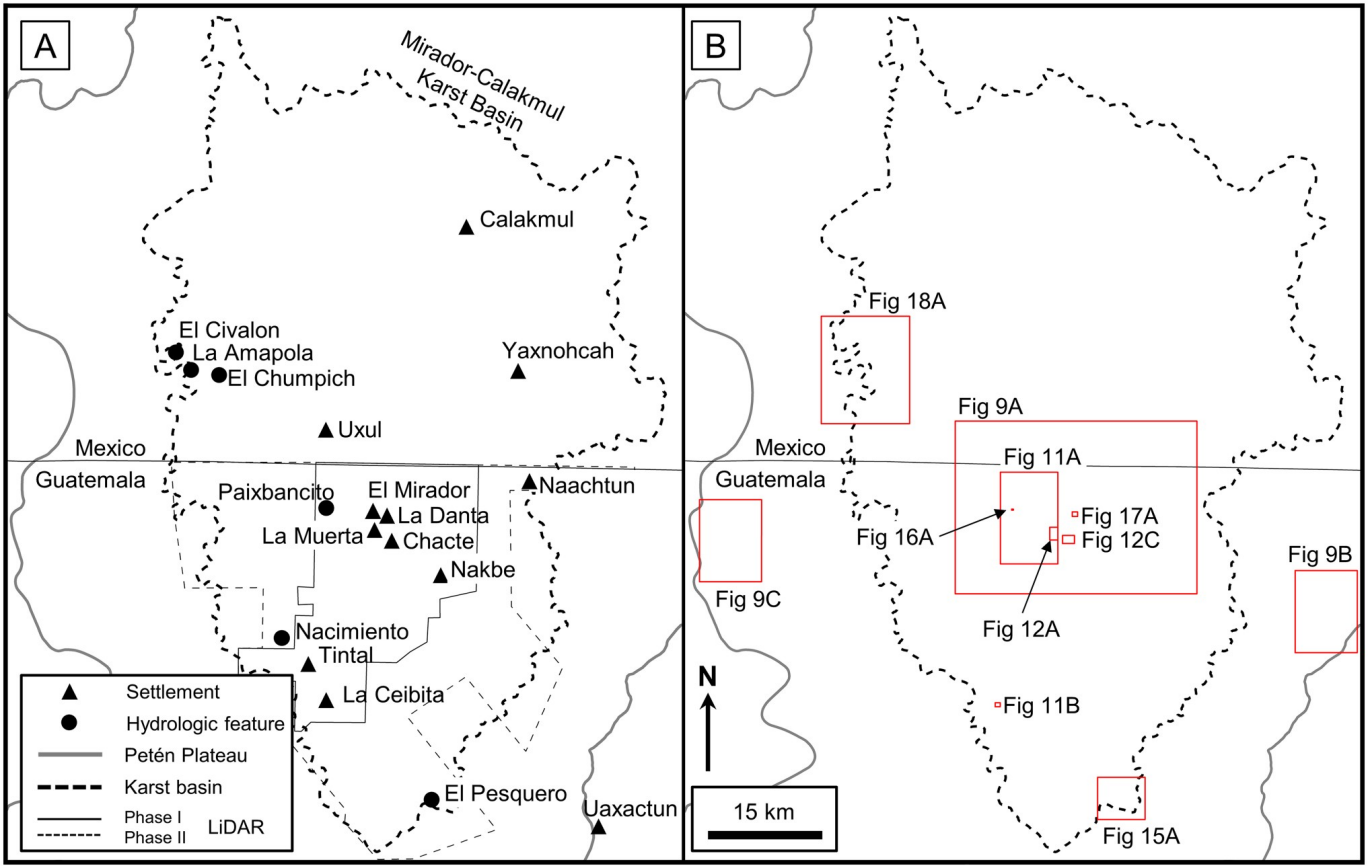
Location map of the study area. (A) Sites mentioned in this paper and the extent of the 2015 Phase I and 2018 Phase II LiDAR surveys. (B) Insets show the location for detailed maps of karst landforms and karst hydrologic features. All layers were produced by the authors and are copyright-free.

Our study encompassed the Petén Plateau along with portions of the RHFZ and the Coastal Plain. Over the course of the project geologic interpretation was performed at multiple scales, dependent on data availability and the scale of features being interpreted. We interpreted geologic structures and karst landscapes over the full extent of the PP (Fig 1). We also interpreted large-scale karst landforms over the study area using satellite data to enable characterization of the Mirador-Calakmul area (Fig 4A and 4B). In addition, the project identified small-scale karst features in the Mirador region throughout an area covered by Light Detection and Ranging (LiDAR) data (Fig 4A). Helicopters were used for aerial survey of selected features and to traverse the heavily forested region. Lastly, we compiled hydrological features over the southern PP and the surrounding wetlands.

### Field methods

During two field seasons in 2018 and 2019, field teams surveyed the karst landscape in the regions surrounding the archaeological sites of El Mirador, Tintal, Nakbe and the intervening areas. Prior to 2019, interpretation of LiDAR data served to identify karst landforms including collapse dolines, subsidence dolines, stream channels, swallow holes, faults, solution corridors, and other large-scale solution features. We up-loaded the GPS coordinates for these landforms to a Garmin inReach Explorer+ to assist in locating them on the ground in the jungle covered environment, and LiDAR images for key areas to a Garmin Montana to allow terrain visualization in dense vegetation while on site. In all, field teams visited over 250 sites during 2018–2019. Some 133 geologic samples were collected from sites that included limestones, dolostones, and gypsum as well as samples from specific features such as cements, brecciated zones, karst karren, and flowstone. Of these, 83 samples were high graded for further analyses. Lastly, all remaining samples are archived in the sample warehouse at El Mirador.

### Laboratory methods

Selection of 28 samples for thin section analysis focused on obtaining rock composition, texture, fabric, and cement to classify them. Core Laboratories Houston Advanced Technology Center analyzed the thin sections. Samples were prepared by first impregnating the samples with epoxy to augment cohesion and prevent loss during grinding. Blue dye added to the epoxy highlighted the pore spaces. The laboratory mounted samples on a frosted glass slide and then cut and ground in oil to an approximate thickness of 30 microns and wedged. They partially stained selected thin sections with alizarin red-S to differentiate calcite (stains red) from clear dolomite (does not stain) and potassium ferricynanide to identify ferroan dolomite (stains medium blue) and ferroan calcite (stains purple). Lab technicians avoided damage of samples containing large amounts of clay by not using stains. Thin section analysis used standard petrographic techniques. Core Laboratories also performed X-Ray diffraction analysis on 8 samples.

### Remote sensing data

Regional visualization and geologic interpretation used elevation data recorded by the Japan Aerospace Exploration Agency (JAXA) [87]. Takaku and others previously reviewed acquisition from the Advanced Land Observing Satellite (ALOS) and processing into a digital elevation model (DEM) [88,89]. Although the DEM was recorded at a horizontal resolution of 5 m, the publicly available data used in the present study, the ALOS World 3D-30m (AW3D30) was resampled to a 30 m resolution. To better understand the vertical accuracy of the data we validated the data using 60 Ground Control Points recorded by the Instituto Nacional de Estadística y Geografía [90,91] and 35 control points for bare earth well pad locations provided by the Guatemalan Ministerio de Energía y Minas. Although the overall Root Mean Squared Error was 5.3 m, close to the nominal 5 m for AW3D30, when classified according to ground cover (bare, low trees or jungle) the error was 3.2 m for bare earth, 5.9 m for low trees, and 9.0 m for jungle cover. This validation indicates that the AW3D30 is not sensing the ground level nor the top canopy in jungle covered areas but a level that is in the upper part of the canopy.

**Fig 5:**
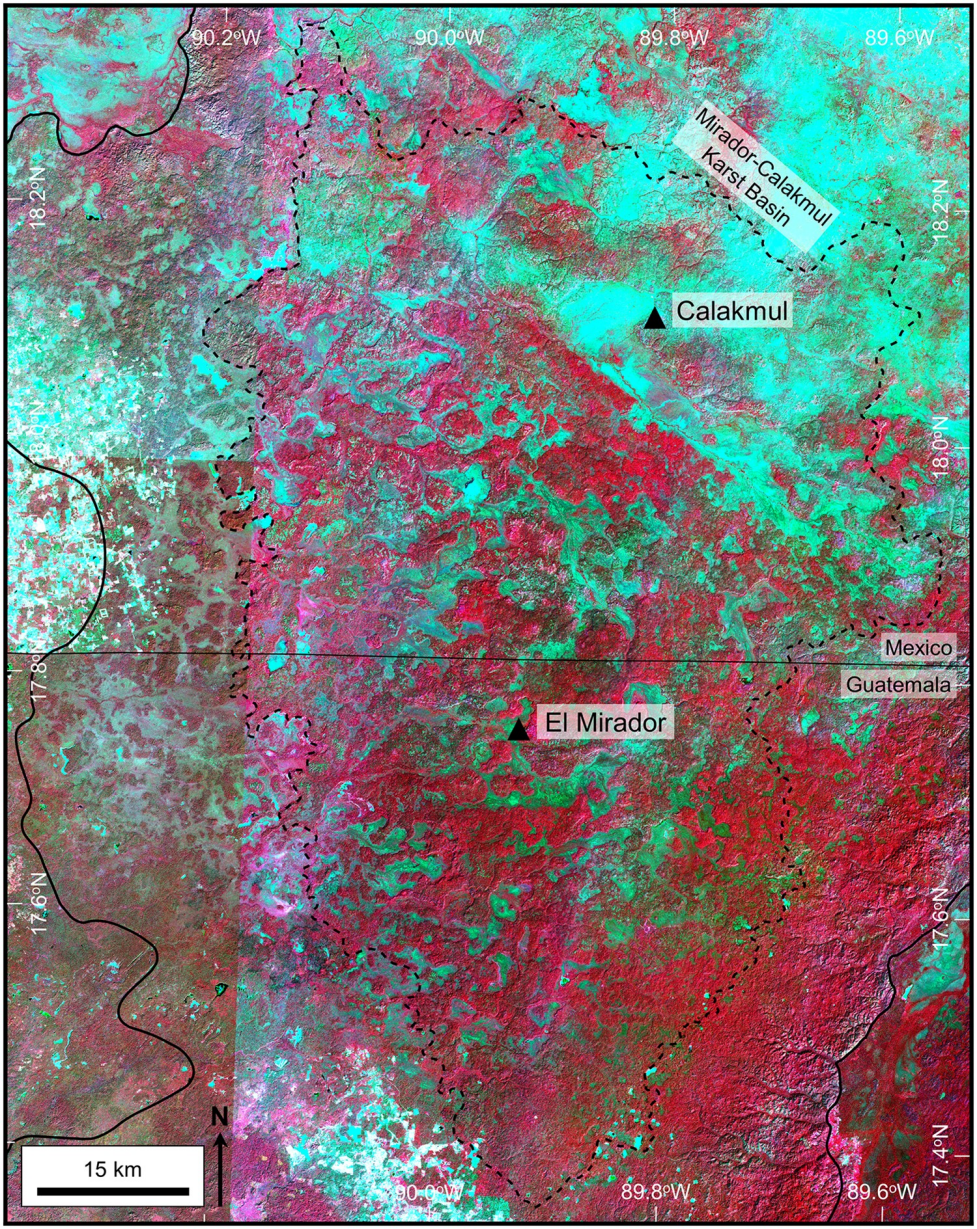
Near-Infrared image of the Mirador-Calakmul Karst Basin. We processed this image from Sentinel-2 multispectral data recorded in early 2017. Tiles along the left side of the image were recorded on different days than the tiles over the central and right side of the image resulting in a visible seam. As well, there is markedly more cyan apparent in the upper right of the image that is not necessarily associated with bajos, due to changes in the dominant upland vegetation. The NIR image shows more detail in vegetation changes and enhances the reflectance of chlorophyll. These data were processed using QGIS software specifically with the Built Virtual Raster tool. We combined near-infrared (B8), red (B4), and green (B3) in a single image to represent upland areas that are shown in reds while bajos are normally displayed in blue-green colors. A large format version of this map is available online as Supporting Information (S1 Map). Sentinel-2 multispectral data have been provided by Centre National D’Etudes Spatiales (https://cnes.fr/en), or CNES, and printed under a CC BY 4.0 license.

Large-scale karst landforms and hydrologic features were imaged using multispectral data recorded by the European Space Agency (ESA) that allowed us to highlight vegetation changes that emphasized karst valleys and *bajos*. Drusch and others previously discussed Sentinel-2 data and reviewed the acquisition and processing of ESA data in detail [92]. Sentinel-2, recorded in 13 bands with its MultiSpectral Instrument, including near-infrared with varying spatial resolution of 10 m, 20 m, and 60 m. We utilized ESA’s True Color Image (TCI) and our own Near-Infrared (NIR) data processed with QGIS software (Fig 5 and S1 Map), both with a 10 m resolution. Downloaded datasets included multiple years during dry periods which maximized vegetation differences [93–96]. The bulk of our analysis utilized data recorded in April 2019, near the end of the dry season, a time with low cloud cover. Data recorded during June 2020, after a heavy rain, served to illustrate stream overflow routes and ponding of water at springs [97].

Visualization and interpretation of small-scale karst features utilized airborne LiDAR data collected by Eagle Mapping starting in 2015 using a Riegl LMS-Q1560 dual-channel system. The Phase I survey covered 645.01 km^2^ centered on the core sites of El Mirador, Nakbe, and Tintal located in the southern MCKB (Fig 4). The survey design included a swath width of 641 m with an overlap of 55%, providing a nominal pulse density of 20 pulses/m^2^. Total pulses were 16.46 billion and total returns were 31.21 billion with a return/pulse ratio of 1.90. The average total return density after processing is 48.39 points/m^2^ and the average ground return density for the survey, as determined after classification with Terrasolid’s TerraScan software, is 2.18 points/m^2^. Internal post-processing with Rapidlasso’s LASTools software included further noise reduction, re-classification, and removal of zero-return areas resulting in an uplift to 2,875,496,652 ground points and a ground density of 5.30 points/m^2^. We created a final DEM using QuickTerrain Modeler, followed by Esri’s ArcGIS software to generate a GeoTIFF raster with a horizontal cell size of 0.5 x 0.5 m. Accuracy of the final dataset is +/- 15 cm horizontally and +/- 30 cm vertically. A Phase II LiDAR survey recorded in 2018 has an average ground return density of 2.95 and increased the total LiDAR coverage to 1703.17 km^2^, covering 87.2% of the Guatemalan portion of the MCKB.

We utilized a variety of other cartographic and geospatial data over the course of the project. Published geologic maps guided our evaluation including the geologic map of Guatemala [98] and multiple individual map sheets by the Servicio Geológico Mexicano [99–103]. We obtained hydrographical data for Campeche modeled by the Instituto Nacional de Estadística y Geografía [104–110], for Petén modeled by the Ministerio de Agricultura, Granadería y Alimentación [111], and a dataset covering the entire YP modeled by the USGS [112,113]. We also acquired a set of 61 orthophotographs covering the Calakmul region [114]. These datasets provided insight into mapping watersheds, overflow streams, swallow holes, *civales*, and artificial reservoirs. Land clearing has obscured the region south and west of the PP in Guatemala making it difficult to identify original extent of natural wetlands. To overcome this obstacle, we accessed a map made by the US Defense Mapping Agency before whole scale deforestation [115].

Esri ArcMap and Open Source QGIS were utilized for visualization, analysis, and interpretation of geospatial data. These included remote sensing data (AW3D30 DEM, Sentinel-2 multispectral, LiDAR), orthophotographs, geological data, hydrological data, and cultural information obtained from the Instituto Geográfico Nacional of Guatemala and the Instituto Nacional de Estadística y Geografía of Mexico. Statistical analyses of doline morphometrics were performed using Rose & Associates Risk Analysis software. The coordinate system used was WGS 1984 UTM Zone 16N.

## Results

### Structural interpretation

We mapped geologic structure across the southern YP to better understand the different structural terrains and their impact on the karst hydrogeology. As indicated above, the PP with its two central anticlines and the Buenavista Escarpment is the most prominent geologic structure in southern YP. The southeast edge of the PP is bounded by the Buenavista Fault [67,98,116]. A seismic line that crosses the escarpment illustrates the normal fault effectively (Fig 2). On this line the MA is a gentle fold of the near surface reflections while the BVF exhibits offset reflections. The Mirador and Calakmul anticlines follow the elevation high from the Carmelita Road to the El Laberinto Bajo, and from the El Laberinto Bajo to the north. This is consistent with previous regional reviews [16,55,68], prior geologic maps in the Calakmul region [74,99,100], and the authors’ observations in the Mirador area. We place the BVF on the east side of the plateau at the base of the sharp topographic break that begins near the Mexico-Guatemala border and extends south beyond Paso Caballos. The BVF marks the western limit of the RHFZ. Normal faults within the RHFZ, many of which show an en echelon or subparallel pattern, illustrate a system of down to the east normal faults. The intervening lows slope west northwest [3,117] indicating a system of half-grabens controlled primarily by underlying structure, not by karst dissolution. On a smaller scale, we interpret normal faults in the Mirador area on LiDAR data, typically located along steep scarps around the large *bajos*.

### Karst interpretation

We interpreted large-scale karst landforms and hydrologic features, present throughout the MCKB and the surrounding areas, using an AW3D30 DEM [87], Sentinel-2 multispectral data [94,95], and ESRI Online World Imagery (Table 1). On the TCI data the low lying *bajos* are dull brown areas juxtaposed against the brighter greener areas representing the upland forests. Dry season datasets highlight this distinction. The mapped *bajos* occur primarily in depressions when compared with the DEM data. The areas digitized show a system of both individual and coalesced *bajos* [118] which follow the KVs. Poljes, closed karst depressions with flat floors and a width greater than 1 km [119], are present sporadically across the MCKB as shown by the DEM data. These depressions typically host a bajo vegetation. We documented their maximum closure with a polygonal outline. Numerous cockpits are present in the south and southeast margin of the PP. They show as sharp depressions on DEM data surrounded by rounded to conical hills. We recorded the cockpits as a point file and the crests of the surrounding hills as a polygonal network that covers much of the region. Residual hills are present along the southwestern margin of the PP and stand out against the level plain on DEM data. the hills present a green upland coloring on the TCI data while the intervening low areas present a mottled brown coloring like that of the *bajos*. We outlined the residual hills to indicate their coverage relative to the flat lying plain. Small lakes, or marshy *civales* common in the upland area along the MA, typically lie in closed depressions observed on DEM data nest within small to intermediate-sized *bajos*. In most cases, open grasslands surround the lakes, which are apparent on the TCI data and Esri Online World Imagery. We delineated these lakes and their surrounding grassland areas as polygons to indicate their relationship to *bajos* on large-scale maps and converted this to a point file to show their distribution for small-scale maps. In many cases, the multispectral data over the *bajos* exhibit vegetation changes which illustrate the patterns of stream channels. We construed these patterns to show the overall drainage pattern. Some of the channels are throughgoing while many, however, appear to flow into closed depressions or dips in elevation. We infer these to represent intermittent streams and swallow holes and captured them accordingly.

**Table 1:** Large-scale karst landforms interpreted on remote sensing data.

Landform	Landscape	Number of landforms	Landscape area (km^2^)	Density (Landforms/km^2^)
Seasonal swamp (*bajo*)	Fluviokarst (MCKB)	861	4533	0.19
Intermittent lake (*cival*)	Upland karst	112	299	0.37
Residual hill (*mogote*)	Karst margin plain	400	2128	0.19
Cockpit	Polygonal karst	2686	2172	1.24

We identified abundant small-scale karst hydrologic features on LiDAR data recorded in the southern MCKB (Table 2). We captured collapse dolines initially as a point file to illustrate their distribution. Later, to enable morphometric analysis, we digitized the perimeter of the collapse dolines in a manner that highlighted the collapse areas and digitized polygons around solution dolines that indicate the maximum area of closure. In the process, we observed numerous breached dolines but did not record them. Intermittent stream channels are present in many of the KVs and *bajos*. Although these were all digitized as line files, we did not capture the countless short arroyos leading from the uplands into the lowland *bajos*. We captured swallow holes along the stream channels as well as those in the closed portions of large *bajos* and dolines as point files to show their distribution. Although not readily apparent on the AW3D30 DEM, we also identified closed portions of the *bajos* and KVs on LiDAR data. Following common usage [120,121] the ponds at Nacimiento and Paixbancito are both considered to be half-blind valleys.

**Table 2:** Small-scale karst hydrologic features interpreted on LiDAR data.

Landform	Number of features	Density (Features/km^2^)
Collapse doline	908	1.41
Solution doline	313	0.49
Intermittent stream	342	0.53
Swallow hole	389	0.60
Closed depression	73	0.11
Half-blind valley	2	0.00

### Hydrogeologic modeling

We compiled and classified hydrologic features, used to model inferring subsurface flow paths, using ESRI Online World Imagery, Sentinel-2 multispectral data, and orthophotographs (Table 3). Google Earth historical images allowed us to understand the presence of water features over time. For this aspect of our hydrogeologic research we worked over a broader area to show the regional nature of groundwater flow patterns. During the project, team members mapped and classified over 4000 individual features in ArcMap. These included wetlands, springs, residual pools, sinkhole ponds, lagoons, intermittent lakes, rivers, lakes, and ponds in half-blind valleys. An unclassified category included all water features not readily categorized. We extracted elevation from the AW3D30 DEM for all features and loaded the resulting dataset into the © Kingdom Software for gridding.

**Table 3:** Hydrologic features identified on the southern PP and surrounding areas.

Feature	Number
Wetland	2468
Spring	261
Residual pool	350
Sinkhole pond	292
Lagoon	206
Intermittent lake (*cival*)	151
River	25
Lake	11
Half-blind valley pond	2
Unclassified	298
**Total**	**4064**

It is important to mention that no anthropogenic features were knowingly used in this study as many have water retention potential aided by clay floors and stone linings. To avoid these, we mapped 714 artificial *aguadas* and reservoirs over southern PP and northeastern Petén. Others may become apparent as more LiDAR data become available over the area. Excluded also were thousands of ponds in cleared fields, most of which are contemporary cattle pond constructions.

Gridding the hydrologic features described above allowed us to create a potentiometric map of the regional water table. Prior hydrogeologists have modeled the groundwater in the eastern YP [39]. Bauer-Gottwein and others synthesized data from multiple regional studies to develop a map of groundwater elevation for the entire YP [43]. These studies utilized data from wells, wetlands, lakes, lagoons, *civales*, and sinkhole ponds all of which they considered to represent the regional water table. Our regional map showing the elevation of the regional water table over the southern PP and surrounding areas provides a more detailed view. As shown by the previous models, the water table slopes east and west from a high under the central plateau anticlines. We recognize that the level of the water table fluctuates from wet to dry seasons. Gondwe and others showed that this fluctuation was as high as 10 m in some areas of Quintana Roo and eastern Campeche [39].

### Lithological analysis

The type locality of the Buena Vista Formation is the exposure on the southern escarpment of the Petén Plateau at Buena Vista Peak, 15 km northeast of Paso Caballos. Wadell first described the section along the escarpment as gypsum interbedded with marls [2]. Vinson described the type section, considered by him to be the lower portion of the formation, as a massive gypsum interbedded with fine-grained limestone, dolomite, and marls [6]. At this location, he measured the stratigraphic thickness of the Buena Vista Fm to be 300 m but indicated that he was not able to include an upper portion of the formation. This gypsiferous portion of the formation is loosely correlative with the Icaiché Formation of Campeche, deposited in a restricted marine environment [7]. A limestone section of the Buena Vista Formation, which conformably overlies the gypsum, has been reported as a grey to light yellow, and fine-grained to crystalline but not described in detail [2]. We will refer informally to this limestone, which crops out over much of the MCKB, as the upper Buena Vista Formation.

**Fig 6:**
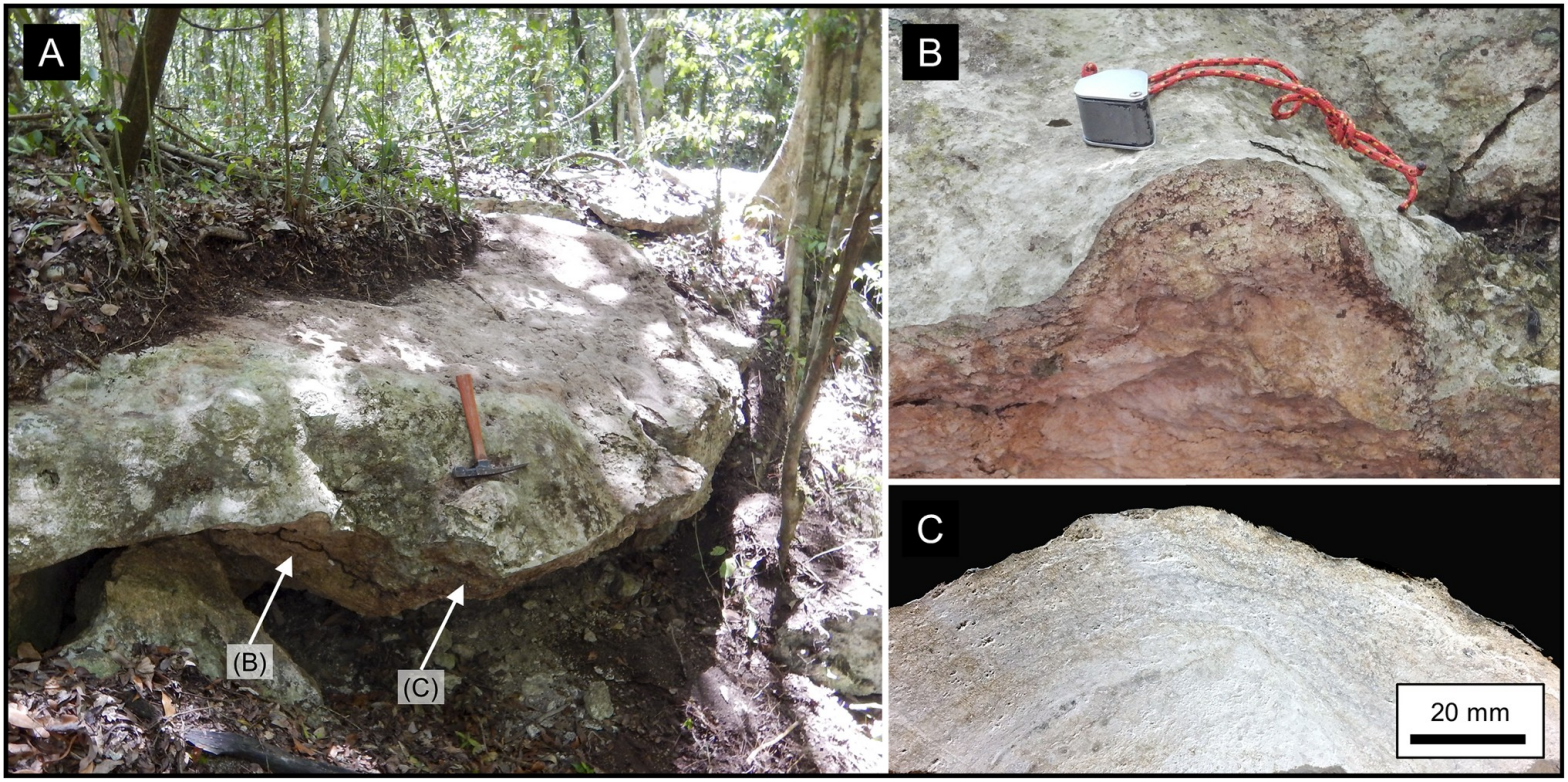
Morphology and fabric of a Buena Vista algal mound. (A) Outcrop of an algal mound in the northeastern rim of Sink 11LM shown in Fig 12A, with a rock hammer for scale. The exposed surface of the mound is a hummocky bedding surface with isolated algal hemispheres on the flanks. (B) Close up photograph of the left-hand hemisphere showing coarse lamination, with a hand lens for scale. (C) Photograph of a slabbed section of the right-hand hemisphere showing crude algal laminations and vuggy pores. The crude layering is formed by an alternation of stromatolitic algal mats and rhizolithic layers.

Within the southern MCKB, where the upper Buena Vista Fm is present, the dominant rock type is an algal boundstone deposited in shallow marine environment subject to repeated exposure. Additional rock types observed include skeletal grainstones, dolostones, and chert in selected exposures. One exposure in the La Muerta upland shows a well-formed algal mound with a hummocky surface and several large algal hemispheres (Fig 6). The 2019 field team observed similar hummocky bedding and evidence of algal mats in multiple outcrops.

**Fig 7:**
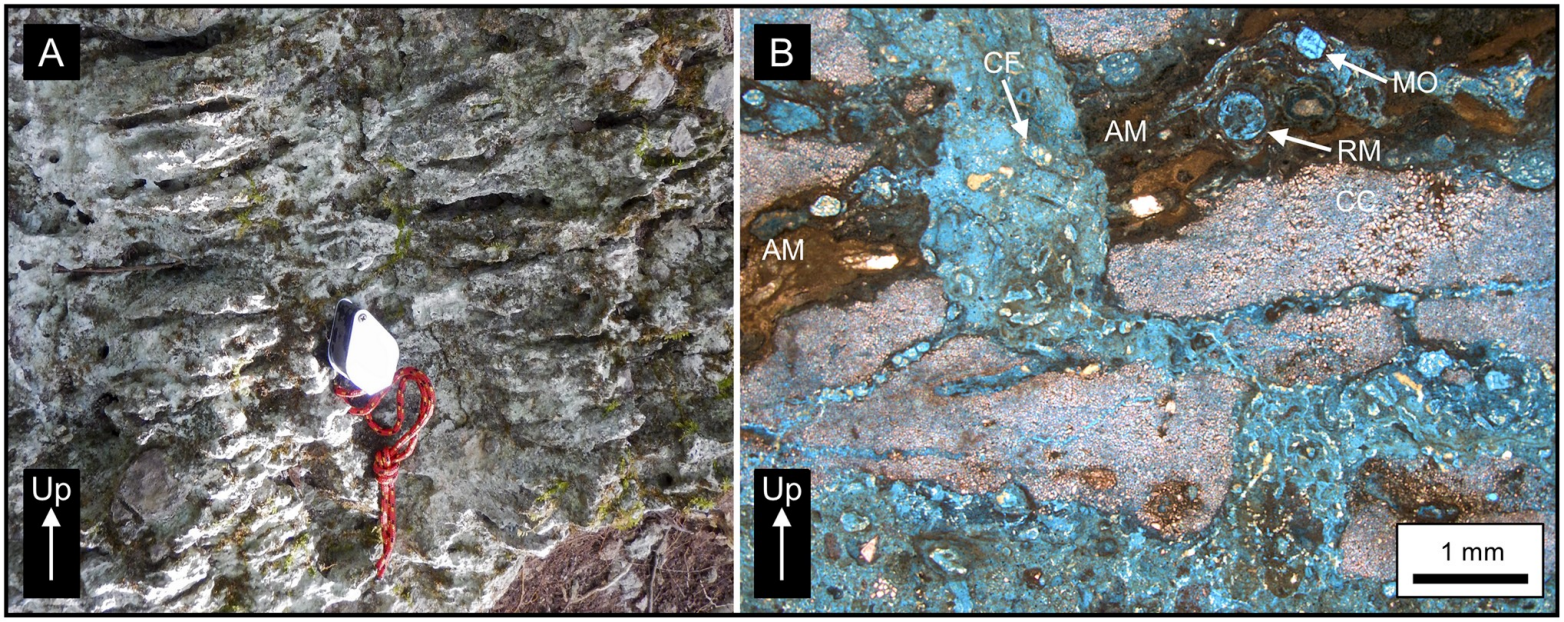
Buena Vista Formation algal boundstone. (A) Outcrop of the Buena Vista Fm in Sink 13LM south of La Muerta, with a hand lens for scale. Erosion of the algal mats has emphasized prominent bedding. The limestone is light grey on weathered surfaces but cream-colored on a fresh surface. The rock is poorly cemented and soft, but exposed surfaces have a harder crust. (B) Microphotograph of sample 19-095B, located in Sink 13LM as shown in Fig 12A. This laminated, pedogenic carbonate consists of wavy laminated, algal/rhizolithic mats (AM) interlayered with microporous, chalky caliche (CC) layers. The algal mats contain common calcified filaments (CF) and root molds (RM). The chalky caliche consists of microcrystalline calcite formed through the pedogenic alteration of limestone bedrock. Traces of limestone intraclasts are present. Root penetration and other pedogenic processes brecciated the caliche crust. Moldic pores (MO) and micropores are common in the algal/rhizolithic mats, and intracrystalline pores are common in the chalky caliche layers.

In thin section, we classify the rock as an algal/rhizolithic boundstone with microbial laminations and large vuggy pores that represent root clasts with minor microspar and rare pyrite cement (Fig 7). The laminated texture is due to the alternation of dense, algal mats and porous, chalky caliche layers. The caliche consists of finely crystalline calcite and is commonly disrupted or brecciated by root penetration and other soil-forming processes. There are abundant biogenic features caused by the pedogenic alteration of the original limestone. Algal boundstones like these have been observed throughout the southern MCKB and are interpreted to represent deposition of algal mats in a shallow marine environment with alternating periods of subaerial exposure and formation of caliche crusts [122]. Caliche lithology and texture, like that observed in our sample, has previously been described from elsewhere in the YP [123]. The interpreted degree of repeated exposure indicated by these rocks coincides with the inference by Gates [68] of a depositional environment on the Yucatán Platform that underwent repeated cycles of rapid eustatic sea level changes during the Eocene [124–126]. Limestone of the upper Buena Vista Fm is soft when first exposed but becomes more indurated with exposure, lending itself well to quarrying and use as building stone by the ancient Maya [127,128].

Mineralogy of select carbonate samples are dominantly limestone with small admixtures of quartz and other minerals. We performed X-Ray diffraction analysis on eight samples collected in the MCKB. Results, shown in Table 4, indicate that for the limestone samples the average percent weight of calcite is 99.16%, while that of quartz is 0.44%. Gypsum, barite, and total clay are minor constituents for a few samples only.

**Table 4:** X-Ray diffraction results for selected Buena Vista Fm samples.

Sample	Lithology	% weight mineralogy from X-Ray diffraction						Whole Rock	Latitude (Dec. Deg.)	Longitude (Dec. Deg.)
		Quartz	Calcite	Dolomite	Gypsum	Barite	Clay			
19–007	Limestone	0.3	99.7	0.0	0.0	0.0	0.0	100.0	17.574048	-89.993404
19-038A	Limestone	0.4	99.6	0.0	0.0	0.0	0.0	100.0	17.579774	-89.999183
19-050B	Dolostone	0.6	38.6	60.5	0.2	0.0	0.0	99.9	17.754266	-89.908856
19–054	Limestone	0.7	97.9	0.0	0.0	0.0	1.5	100.1	17.753317	-89.906882
19-060A	Limestone	0.6	99.4	0.0	0.0	0.0	0.0	100.0	17.749814	-89.896687
19–075	Limestone	0.4	99.6	0.0	0.0	0.0	0.0	100.0	17.749406	-89.920205
19-114A	Limestone	0.5	99.0	0.0	0.0	0.5	0.0	100.0	17.680972	-89.832738
19-179A	Limestone	0.2	98.9	0.9	0.0	0.0	0.0	100.0	17.677489	-89.834799

## Discussion

### Karst landscapes of the Petén Plateau

**Fig 8:**
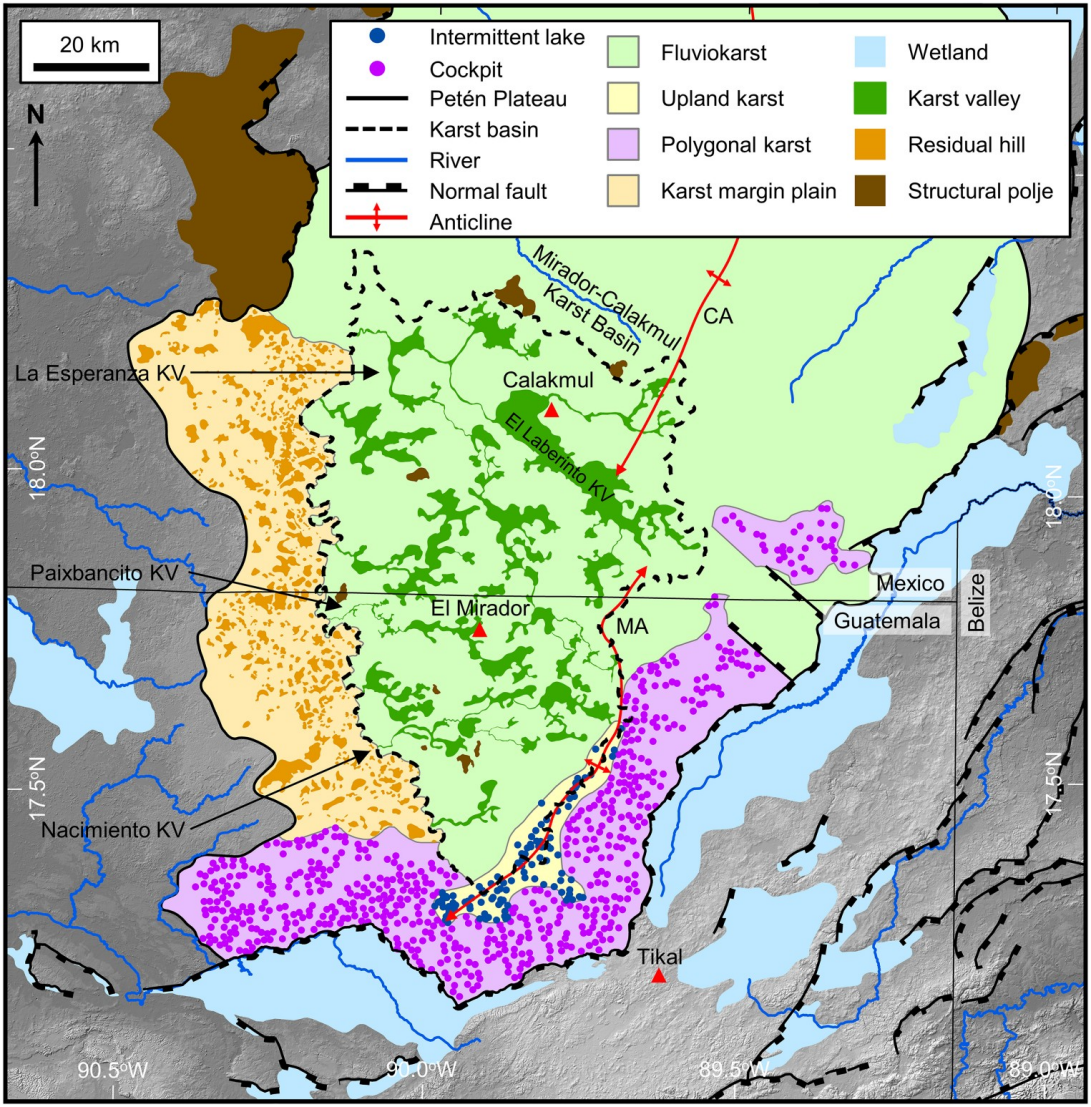
Karst landscapes of the southern Petén Plateau. We define each of the landscapes, four of which are shown in this map, from their primary karst features as follows: (1) Fluviokarst is a landscape that is transitional from fluvial to karst dominated. The most common karst landform in terms of areal coverage are the karst valleys, digitized in a manner that shows the connectivity of the valley system but does not necessarily imply complete fluvial drainage. This map shows the largest of these, but not all. Modeled drainage [113] indicates a dendritic pattern of intermittent streams overprinted by karst depressions. Other karst landforms include structural poljes, collapse dolines, and solution dolines. (2) Polygonal karst is a terrain dominated by cockpits and cone-shaped hills mapped as a polygonal mesh. At this scale we note the cockpits rather than their bounding polygons, here decimated to about 20% of the total number. Other landforms present include intermittent lakes, *bajos*, and caves or collapse features occurring in cockpits [129]. (3) The karst margin plain is a region marked by residual hills that remain on a surface planated by karst activity. The area has expanded through corrosion planation which includes the vertical dissolution, lateral undercutting of hills by swampy zones, and lateral expansion through spring sapping. (4) The upland karst, centered on the broad MA in the southeastern Petén Plateau, is a landscape characterized by the presence of intermittent lakes in a region of poor surface drainage. The PP is bounded by normal faults on the southeast and northwest which served to create closed structural poljes and low-lying wetlands. AW3D30 elevation data have been provided by JAXA (https://www.eorc.jaxa.jp/ALOS/en/aw3d30/) and printed under a CC BY 4.0 license. All other layers were produced by the authors and are copyright-free.

Karst landscapes dominate the geomorphology of the Petén Plateau, which varies from rolling hills interspersed with shallow flat-floored depressions in the central plateau to rugged hills with deep steep-sided valleys in the southeast. The plateau, with a total area of 27,000 km^2^, has five different landscapes, each dominated by a single karst landform (Fig 8 and S2 File). A fluviokarst landscape covers the bulk of the plateau (Table 5) in which the geomorphology is transitional from a fluvial to a karst terrain dominated by KVs. Dominating the south and southeast margins is a polygonal karst landscape with a network of residual hills and cockpits dissected by KVs. Along the southwest flank is a karst margin plain, a mature karst region denuded to form an alluvial plain with residual hills. A small upland karst in the southeast of the plateau, exhibits numerous intermittent lakes along the crest of the Mirador Anticline. The northern portion of the plateau is a landscape consisting primarily of base-level poljes. Lastly, structural poljes and wetlands flank the plateau along all sides.

**Table 5:** Aerial extent of karst landscapes of the PP.

Landscape	Area (km^2^)	% Plateau (km^2^)
Fluviokarst	16,980	63.1
Polje karst	5,349	19.9
Polygonal karst	2,172	8.1
Karst margin plain	2,128	7.9
Upland karst	299	1.1

Assemblages of karst landforms make up karst landscapes, where a dominant landform is typically more common than others [8]. Variations in lithology, underlying geologic structure, depth to groundwater, and surface drainage control the nature of a landscape and its boundaries. Karst develops through dissolution of carbonates and evaporites by acidic water [130]. Rainwater can form a weak carbonic acid through combination with carbon dioxide (CO_2_) in the atmosphere and may be further acidified through combination with soil CO_2_ and organic acids produced in the uppermost soil layers [8,119,120]. Karst exhibits the most significant variation in landforms and the highest denudation rates in the humid tropics. Fluvial erosion of the central YP began during initial uplift in the Late Paleogene. As surface erosion continued, water entered the subsurface through joints, fractures, and faults and began to develop subsurface pipe-like conduits through dissolution. Given the extent of limestone, gypsum, and dolomitic rocks, the tropical climate, and high precipitation the PP has experienced significant karstification since its uplift leading to a variety of landscapes.

**Fig 9:**
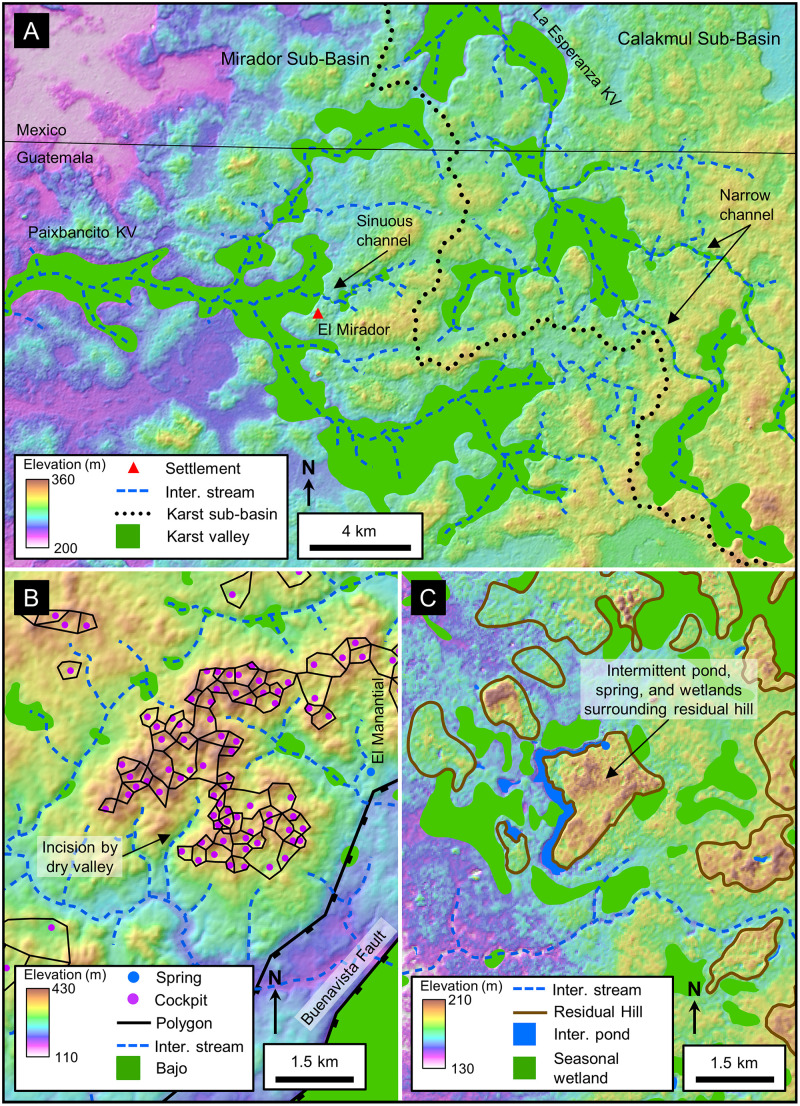
Detailed views of fluviokarst, polygonal karst, and karst margin plain landscapes. (A) Interpretation of the fluviokarst terrain displayed with two major karst valleys (KV), Paixbancito KV and La Esperanza KV. We interpret the narrow channels, sometimes sinuous, connecting broad amorphic depressions as ancestral fluvial channels. In this figure we illustrate intermittent streams to show the throughgoing nature of each valley system instead of channels punctuated by swallow holes as detected on LiDAR data. (B) In the polygonal karst landscape cockpits are more dense and deeper along the Buenavista Escarpment, suggesting that their formation is associated with uplift of the plateau. The polygonal mesh connects the summits of cones surrounding each cockpit. Dry valleys incise the cockpit terrain implying a younger age of valley erosion. Small *bajos* are present in the dry valleys, and at least one spring is present near the BVF. (C) Denudation of the karst margin plain has created residual hills with intermittent ponds typically on their steep flanks, a sign of spring sapping. Wetlands cover a large portion of the plain. Stream channels are absent, apart from intermittent streams that emanate from the MCKB, cross the karst margin plain, and drain to rivers further west. AW3D30 elevation data have been provided by JAXA (https://www.eorc.jaxa.jp/ALOS/en/aw3d30/) and printed under a CC BY 4.0 license. All other layers were produced by the authors and are copyright-free.

The southern core of the PP is a fluviokarst landscape of subsurface drainage that is transitional from fluvial to karst (Fig 9A). Common landforms include karst valleys, poljes, and dolines superimposed on a relic fluvial drainage pattern. The transition from a fluvial valley to a KV takes place as the valley becomes underdrained because both the valley and its tributaries lose their flow to the developing system of subsurface drainage conduits [8]. Gates provided an early view of the central Petén landscape with his description of KVs, often occupied by seasonal wetlands, having an inherited fluvial pattern [16,68]. Heraud-Pina mapped fluviokarst systems, and KVs, in portions of the Arroyo Desempeño on the west flank of the PP and the Escondido watershed on the east flank of the plateau highlighting the presence of a shallow subsurface drainage [17]. Fluviokarst has also been identified in the southern plateau by Marshal who noted the poorly developed surface drainage and abundant sinkholes that drain into an extensive network of solution fractures, and by Day who described a system of dry valleys with ephemeral streamflow [5,131]. More recently, the fluviokarst landscape of the MCKB has been mapped in detail using satellite, LiDAR, and other remote sensing data and shown to include a variety of karst landforms including dry KVs, solution and collapse dolines, half-blind valleys, hanging valleys, solution corridors, sinking streams, and swallow holes that overly a relic dendritic drainage with occasional meandering channels [37]. The MCKB is exceptional in having a fluviokarst landscape covering over 4,500 km^2^ with 1,300 km^2^ of seasonal swamps (Table 6) contained within in a system of karst valleys.

**Table 6:** Wetlands in karst landscapes. Wetland area shown is the aggregate of either *bajos* in the fluviokarst and polygonal karst terrains, wetland areas in the karst margin plain, or the combined *bajos* and *civales* in the upland karst terrain.

Landscape	Area (km^2^)	Wetlands (km^2^)	% wetlands
Karst margin plain	2128	1675	78.7
Fluviokarst (MCKB)	4533	1284	28.3
Upland karst	299	35	11.7
Polygonal karst	2172	39	1.8

The southeastern margin of the PP is a polygonal karst terrain (Fig 9B), an inherently rugged area in which enclosed depressions, or cockpits, are surrounded by conical hills whose summits can be connected by lines to produce polygonal patterns [132]. Although there is an extensive literature regarding karst areas dominated by either cockpits or cones, Jones and White noted a continuum between landscapes dominated by depressions or residual hills and that the concept of polygonal karst covers the full range [133]. Day described polygonal karst from La Libertad Anticline, south of Flores [134]. Although individual karst landforms (caves, cones, dry valleys, *mogotes*, solution corridors) of the southeastern plateau have been noted [4,5,39,135–137], the terrain has previously been classified as polygonal karst [131,138]. Our interpretation of the cockpits observed in the AW3D30 DEM enabled us to map a patchwork of polygonal nets of their surrounding hills. KVs with intermittent streams dissect these networks. This dissection by KVs indicates a polyphase history in which development of the polygonal karst was associated with uplift and exposure of the plateau flank (49–5.3 Ma), followed by more recent development of the KVs after extension of the RHFZ created the Buenavista Escarpment (5.3–1.8 Ma). The dissection may have been further enhanced during multiple sea level low stands of the Late Pleistocene which lowered the regional water table throughout the YP and lead to the formation of the cave systems in the Yucatán and Quintana Roo through aggressive karst dissolution [5]. Because of rapid drainage into the cockpits, *bajos* are scarce in the polygonal karst creating a terrain with a low percentage of wetlands (Table 6). It is this 10–15 km wide polygonal karst, a rough landscape that would have hindered trade and communications, that is referred to as a buffer zone between the Calakmul and Tikal polities during the Classic period [21,77].

A karst margin plain, a region of residual hills on a backdrop of an alluvial plain (Fig 9C), dominates the southwestern margin of the PP and expands eastward through time via spring sapping. Jennings defined a karst margin plain on the flank of a karst terrain with frequent residual hills on a bedrock plain covered by alluvium [120]. Long-term denudation lowers such plains and neighboring valleys merge to become a broad wetland subjected to unconfined runoff during the rainy season [8,119,139]. Steepheads embay the margins and the plain expands into the karst terrain through spring sapping and undercutting of the hillsides [120,140]. McDonald has described a karst margin plain on the northeast side of the Maya Mountains in Belize where the residual hills take the form of karst towers [141,142]. Here, the residual hills often have footcaves, or solution notches developed by the spring action, accompanied by intermittent ponds that are locally sourced. Gerstenhauer detailed a karst margin plain in Tabasco flanking a region of polygonal karst along the leading edge of the Chiapas Fold and Thrust Belt [143]. Here, the residual hills, regionally known as *mogotes*, are 2–10 m high with common footcaves and marshy ponds at their base. The plain has extensive wetlands after large rains and expands as the steep slopes above the footcaves collapse. In the plain along the southwestern flank of the PP, residual hills are up to 40 m high. Ephemeral marshy areas and ponds surround the hills, as observed on aerial imagery and remote sensing data. Denudation of the plain over time has resulted in the merger of neighboring KVs creating a region that is subject to unconfined flow during the rainy season creating widespread seasonal wetlands (Table 6). Steepheads and springs are present on the east flank of the plain cutting into the more elevated fluviokarst terrain and leaving a low line of hills which march progressively eastward through time.

Nestled between the polygonal karst and the fluviokarst landscapes of the southern PP is an upland karst landscape area with intermittent lakes or ponds. Upland karst landscapes, low relief areas with doline karst and ephemeral lakes, ponds, and marshes have been described from the Burren Plateau in Ireland [144,145], the Glasgow Upland in Kentucky [28], and the Mitchell Plain of Indiana [146]. Intermittent lakes in the upland karst of the PP are present in a 5 km wide strip along the crest of the MA, a broad fold with low dips to the west and east. Surface relief in this area is low and KVs are absent. Intermittent lakes and ponds nest within small to intermediate size *bajos* creating a moderate percentage of wetland areas (Table 6). Most of the karst lakes and ponds identified have water in them at the present time. A few have only grasslands present at this time, but they we view them as ephemeral given that water is often present on historical Google Earth images. The fill material of these upland intermittent lakes has not been sampled through coring or trenching. Given this, we speculate that the trapping mechanism for these small lakes is periodic plugging of the conduit system by organic debris and clay followed by occasionally flushing.

The northern portion of the PP is a landscape dominated by base-level poljes. These are large-scale depressions in which dissolution has lowered the surface to the regional epiphreatic zone [119]. Heraud-Pina previously mapped this landscape as part of a larger region that extended off the plateau to the northwest [17]. Most of the depressions, with a mean area of 17.97 km^2^, have wetland areas at their lowest point, typically along a faulted margin.

### Mirador-Calakmul Karst Basin

**Fig 10:**
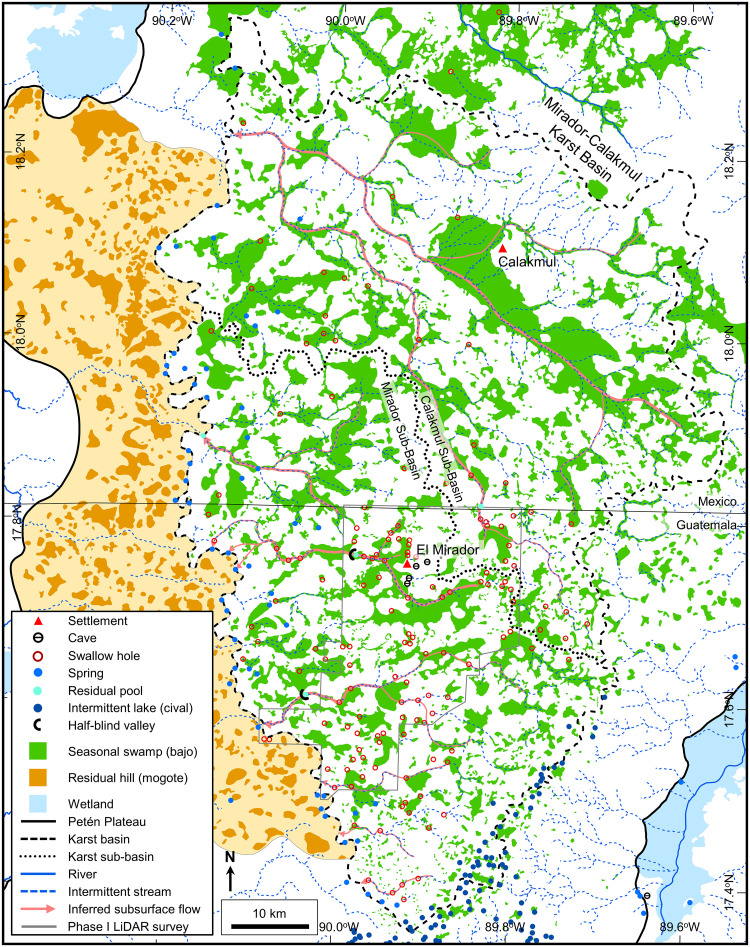
Drainage map of the Mirador-Calakmul Karst Basin. Key hydrologic features are shown as follows: (1) We interpret seasonal swamps, or *bajos*, in a manner that shows the connectivity of the overall system of karst valleys and yet indicates where select *bajos* have little or no drainage. (2) Collapse dolines and solution dolines, both interpreted on LiDAR data, are too numerous to illustrate at this map scale. (3) We illustrate intermittent lakes, or *civales*, in the uplands along the southeastern margin as points rather than the actual polygons to better exhibit their distribution. (4) Within the LiDAR survey we only interpret intermittent streams where there is a dry channel, but we have decimated their display because of the map scale. Outside of the LiDAR survey we assume that intermittent streams follow the major dry valleys. (5) Within the LiDAR we recognize swallow holes as discussed but only show 20% here because of the map scale. Outside of the LiDAR survey we infer swallow holes from the DEM data along the course of the intermittent streams. (6) Caves shown in the MCKB are solution pockets, overhangs, and other collapsed features. (7) Traditionally, hydrogeologists illustrate perennial underflow springs and intermittent overflow springs with different symbols. Without field verification of spring flow in the MCKB we show all springs with a solid blue circle. In the interior of the basin, we identified points with standing water other than anthropogenic *aguadas*. Although many of these may be intermittent overflow springs, we did not classify them as springs without field verification. (8) Hydrologic modeling demonstrates that regional groundwater flow within the MCKB is westward. Flow paths, necessarily inferred, underdrain the major karst valleys and exhibit a dendritic pattern. (9) Basin boundaries are illustrated with a dashed line implying that the boundary is not exact, and sub-basin boundaries are shown with a dotted line indicating the boundary of a smaller unit. (10) We identified residual pools in four locations along intermittent stream channels. Two of these, Paixbancito near El Mirador and Nacimiento near Tintal are at the closed end of half-blind valleys. In the case of Paixbancito the closing floodplain berm is 2 m high and has three distinct overflow channels. (13) Wetlands outside of the MCKB are present throughout the karst margin plain and in low lying regions on the flanks of the Petén Plateau. Exsurgence springs feed these wetlands along the west margin of the MCKB and on the flanks of the Petén Plateau. A large format version of this map is available online as Supporting Information (S2 Map). All layers were produced by the authors and are copyright-free.

The Mirador-Calakmul Karst Basin exhibits all the karst hydrologic features common to karst basins in addition to inferred groundwater flow routes and well-defined boundaries. Following the present interpretation, the basin covers an area of 4533 km^2^. Ground elevation in the basin reaches 406 m on the southeast margin, 375 m on the north margin, and 250–300 m along the western margin providing a gentle slope to the northwest. A drainage map of the MCKB illustrates the key hydrologic features and other components of the basin described below (Fig 10, S2 Map, and S3 File).

#### Seasonal swamps

Although not present in all karst terrains, wetlands in the form of swamps or marshes are normally present in poorly drained karst regions [29,147]. In a fluviokarst setting, such as the southern Petén Plateau, the transition from a fluvial valley to a KV takes place as the valley becomes underdrained because both the trunk and tributary streams lose their flow to the developing underground drainage system [8]. KVs have wide valley floors developed on limestone, often riddled with dolines, and as a whole form elongated depressions. They are commonly bounded by steep sides or scarps that evidence collapse of the valley floor [148,149]. In a fluviokarst landscape, KVs may display varied terrains where the upstream reaches maintain much of their fluvial character while the lower reaches consist of aligned closed depressions [150]. Because the overall drainage is poor, clay soils seal the bottom of the depressions leading to the development of swamps [120].

**Fig 11:**
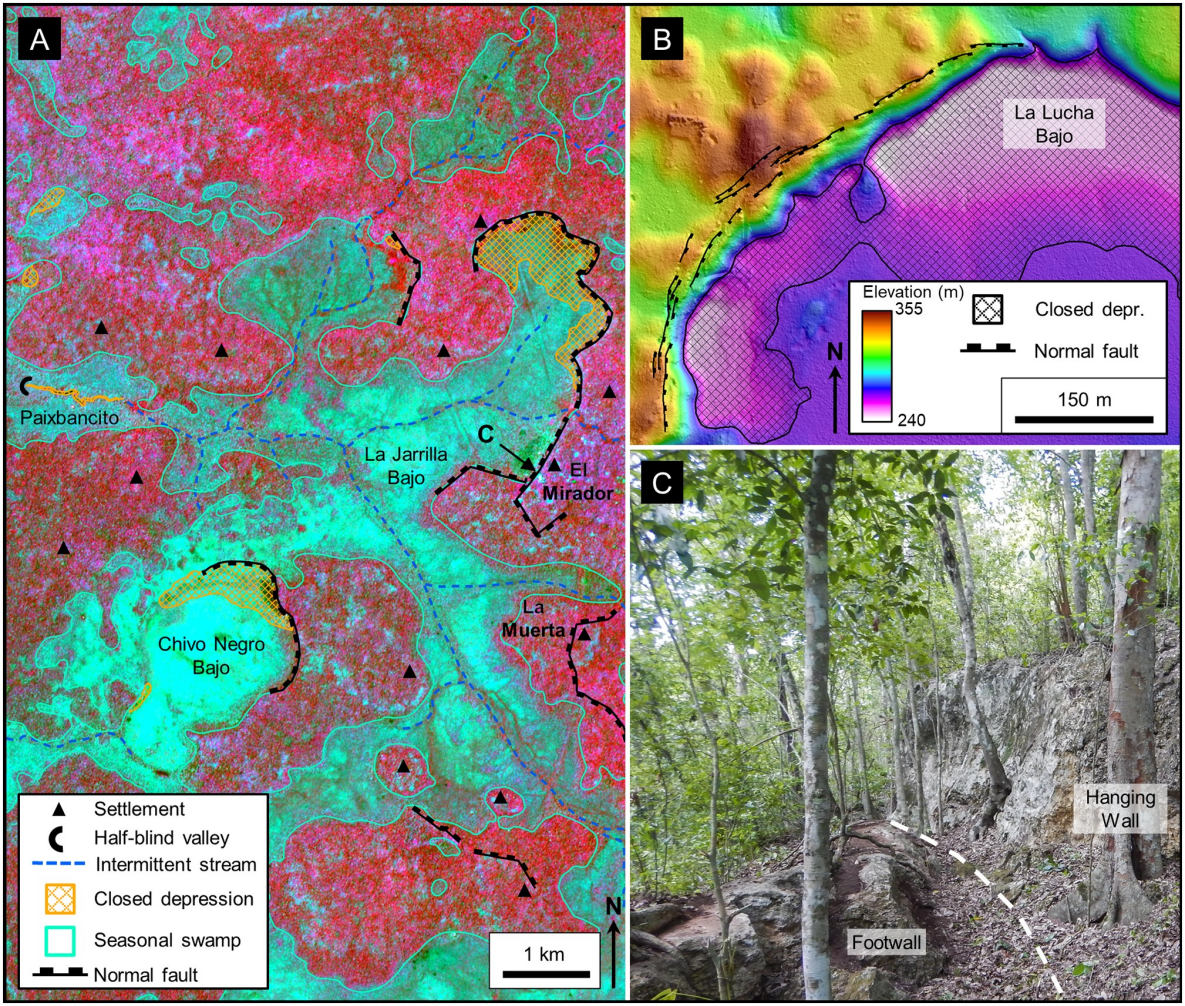
Seasonal swamps, or *bajos*, in a karst valley. (A) Sentinel-2 NIR image covering the region west of El Mirador. In this image the upland areas have a deep red color and the lowland areas, or *bajos*, have a dark blue to cyan color. Several of the individual *bajos* are bounded by faults indicating that their origin is, in part, related to structural collapse. In many cases the faults have generated closed depressions, such as in La Jarrilla and Chivo Negro *bajos*. Our interpretation has identified over 70 closed depressions of this nature on LiDAR data in the southern MCKB. Intermittent streams connect the *bajos* in this view into a larger karst valley. Large storms overwhelm the swallow holes, and the streams drain slowly to Paixbancito, a residual pool in a half-blind valley. (B) We interpret faults on LiDAR data by direct observation of offsets as seen in this image of the bajo margin southwest of La Ceibita. In this case, they form an en echelon fault system. Here, collapse of the footwall has created a closed depression in the La Lucha Bajo. In other cases, chains of collapse dolines may indicate fault zones in the subsurface, as observed on the flanks of the La Muerta upland. (C) Photograph of a fault on the west side of the Mirador upland, flanking the La Jarrilla Bajo. A dashed white line represents the surface trace of the fault. Offset of the fault is 3–4 meters and 2–3 cm of flowstone coats the exposed scarp. At select locations along the outcrop a brecciated zone is present in the hanging wall. To the left of the footwall block in the picture is a second, parallel fault. Similar brecciated fault zones are present in the field on the south side of the Nakbe upland and on the north side of the Tintal upland. Sentinel-2 multispectral data have been provided by CNES (https://cnes.fr/en) and printed under a CC BY 4.0 license. LiDAR data have been provided by FARES (https://www.fares-foundation.org/) and printed under a CC BY 4.0 license.

Seasonal swamps, or *bajos*, dominate the MCKB covering 28% of the basin (Table 6) and in selected areas reaching as much as 40–50% of the land surface (Fig 11A). The literature has commonly referred to them as karst features such as dolines and poljes but has rarely defined them in a geologic sense. Ecologically, the *bajos* are lowlands with a unique set of vegetation dominated by inkwood trees (*palo tinto*). In the overall fluviokarst landscape of the basin, *bajos* may form in KVs, poljes, or individual solution dolines. Gates, in his description of the geomorphology of the Calakmul Biosphere Reserve, noted that the large *bajos* were once fluvial valleys but are now seasonal wetlands [16]. Previous articles have described the seasonal nature of this wetland system [49,50,151]. Drainage of the extensive *bajo* system is slow for several reasons including flat floors with slopes of only 1–2 degrees [152,153], half-blind valleys, and inefficient karst drainage in closed depressions [5]. The large *bajos* enclose depressions flanked by fault scarps (Fig 11B and 11C) and contain swallow holes. Over 70 closed depressions formed through the coalescing of collapse dolines, solution dolines, and poljes have been mapped in the KVs on LiDAR data, with a mean area of 199,018 m^2^. During heavy rains in the wet season, water fills the closed depressions, overwhelms the drainage conduits, and stagnates to form the seasonal swamps where it either evaporates or drains slowly into the subsurface.

We evaluated the extent and distribution of the *bajo* system of the Mirador-Calakmul region showing a fluviokarst drainage pattern of KVs along with more isolated *bajos* in solution dolines and poljes. Prior authors have mapped the *bajo* system using aerial photos, topographic data, and remote sensing data [32–34,36,154], but have not covered the full extent of the basin. Turner and others classified Landsat data on the Mexican portion [155] of the MCKB while Magee did the same for the Guatemalan portion [156] of the MCKB. Although this methodology may be reasonable to determine the area covered by specific vegetation types, the maps generated do not illustrate the drainage system of the KVs. To better understand the karst nature of the basin and drainage routes, we mapped the *bajo* system of the southern PP. Fig 10 shows the distribution of *bajos* throughout the MCKB. All the KVs drain to the west and northwest and exit the western edge of the basin.

#### Dolines

Dolines, also known as sinkholes, are circular to sub-circular hollows a few meters up to 1 km in diameter, typically formed in karst environments, that serve to recharge water into the subsurface [119]. In cross section they may be bowl-shaped and shallow to funnel-shaped and several hundred meters deep. These depressions form through various processes including collapse and dissolution. In an ideal sense there is a distinction between a solution doline created by dissolution of limestone and a collapse doline created by collapse of a cave or void. In practice, however, it is not always possible to define the exact origin and many dolines are the result of a combination of collapse and dissolution. Alignment of dolines occurs through enhanced dissolution along joints or faults [157]. In a karst basin, dolines function to drain water rapidly into the subsurface conduit network though vadose conduits, fractures, swallow holes and small caves [11,46,158,159].

**Fig 12:**
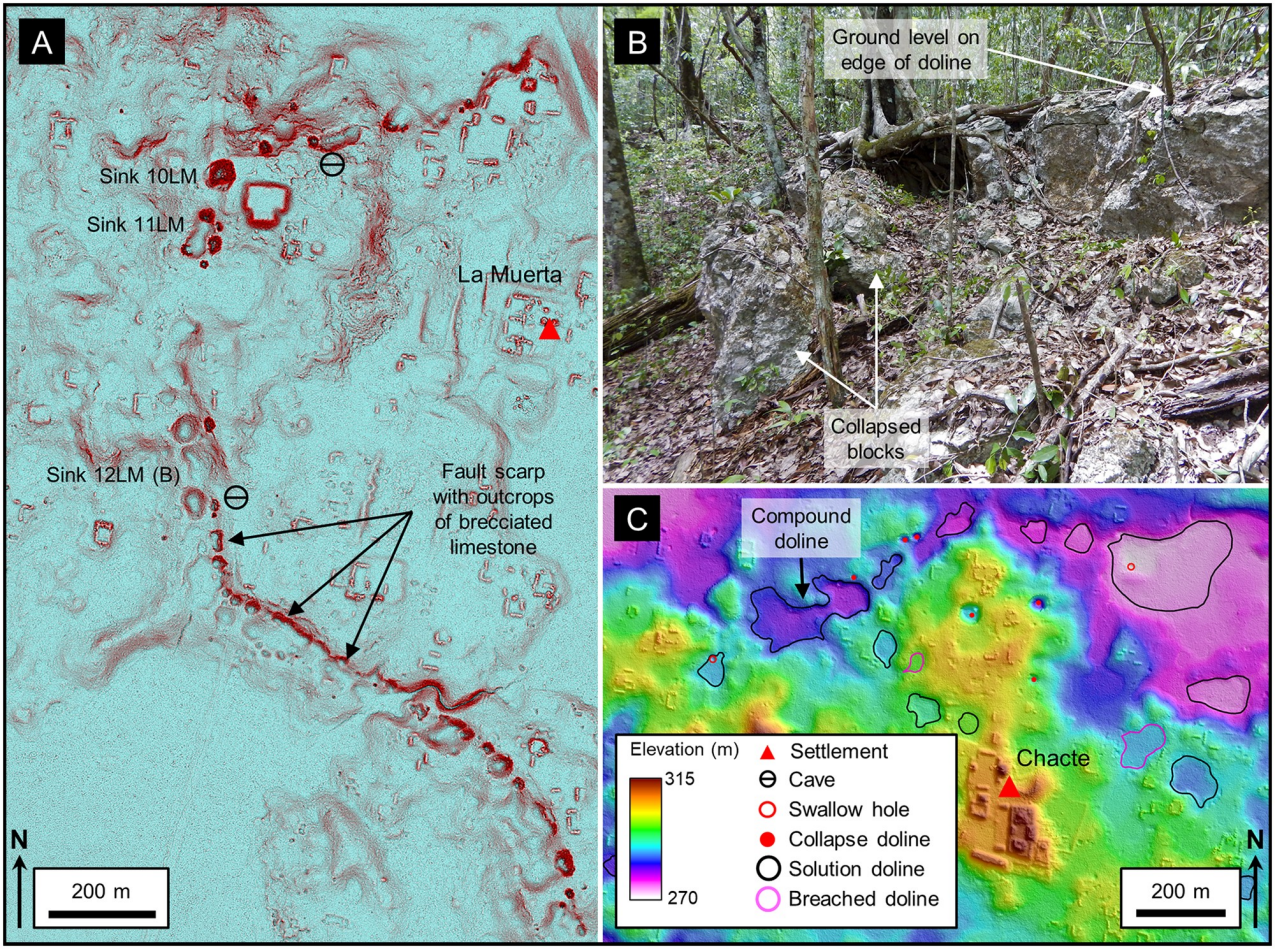
Collapse and solution dolines. (A) Red Relief Image Map (RRIM) showing a line of collapse dolines that flanks the La Muerta upland. The image is a combined visualization of the topographic slope, concavities, and convexities that emphasize large and small features in a single image [167], processed using QuickTerrain Modeler. Many of the dolines have small overhangs, steep scarps, a V-shaped profile, and large boulders scattered along the doline floor, all indicative of their collapse origin. More substantial collapsed caves are present in two of the dolines. Most of the dolines are circular in form, but a few are oval shaped with the long axis parallel to the alignment. A few of the dolines, such as Sink 11LM, are compound dolines where two or more depressions have coalesced over time. Sink 10LM has a scarp 3 m high on the northeast side where part of a temple platform has collapsed, indicating activity younger than the structure. Along the south and southwest flank there is a gentle scarp 5–10 m high with multiple outcrops of brecciated limestone. We interpret this line of collapse dolines and the weathered scarp to indicate that there are deep-seated normal faults that frame the high block, form a locus of dissolution, and serve to down drop the *bajos* to the north and south. (B) Collapsed blocks on the eastern rim of Sink 12LM. The two blocks have detached from the doline rime and slid downslope. Other large limestone blocks are present on the slopes of the doline from the rim to the floor of the depression. (C) LiDAR data from the nearby Chacte area show that solution dolines have a more random distribution, although the dolines along the northern edge of the upland appear to loosely align along structural trends. Swallow holes drain several of the dolines. Two dolines exhibit channels where fluvial erosion has breached the formerly closed depressions. LiDAR data have been provided by FARES (https://www.fares-foundation.org/) and printed under a CC BY 4.0 license.

**Fig 13:**
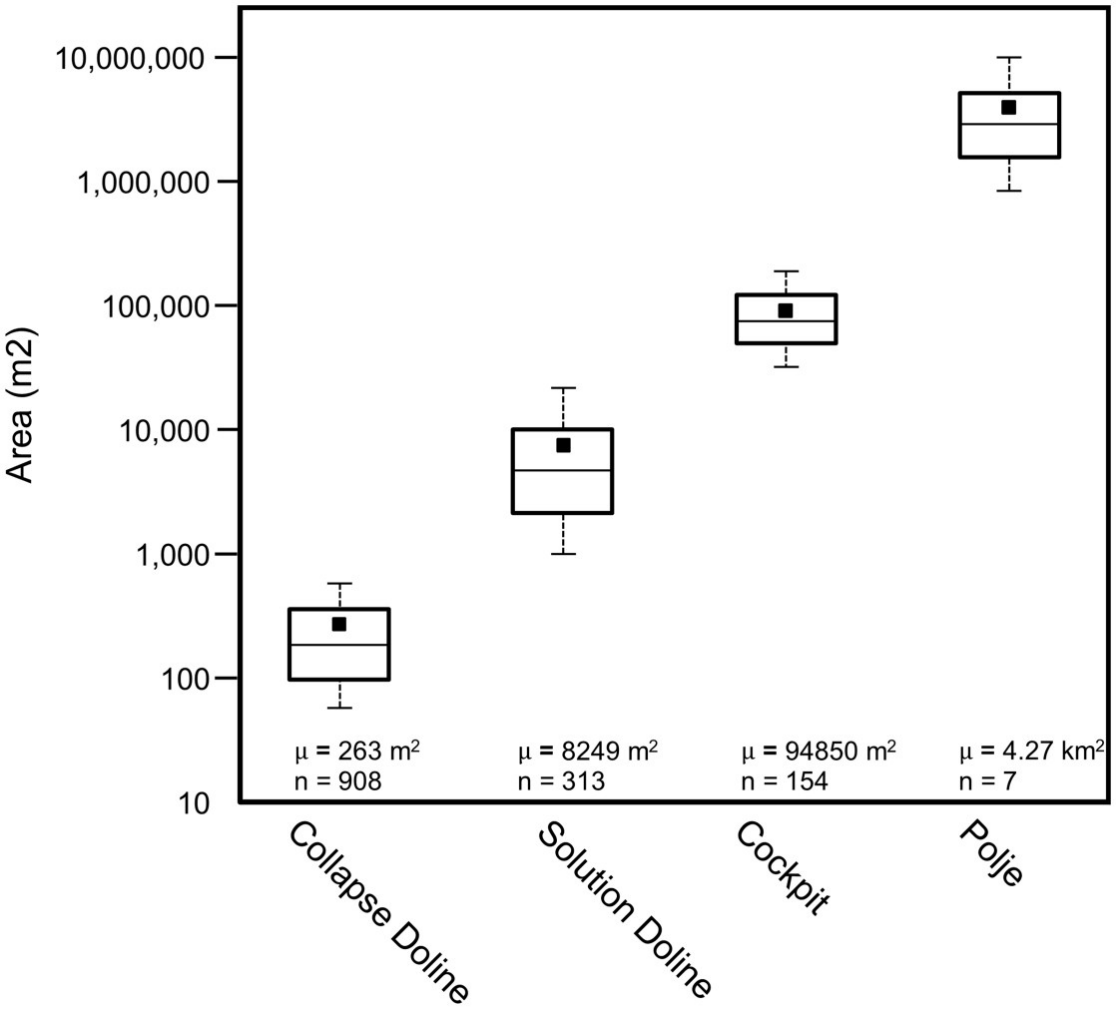
Box plots showing the area of karst depressions. The boxes indicate the 25th and 75th percentiles, the whiskers show the 10th and 90th percentiles, the square shows the mean, and the horizontal line indicates the median. For the collapse dolines and cockpits, the area measured is that of a perimeter which circumscribes the portion of the slope influenced by the doline [168,169]. This dimension is particularly important for dolines that occur on a slope with upslope slumps that may lie outside of the closed depression. For solution dolines and poljes, the area measured is the maximum closed area. Statistically, there is no overlap in area between the P10/P90 ranges of the four types of depressions. We only considered poljes in the fluviokarst landscape of the southern PP for this analysis. Base level poljes in the northern PP, and structural poljes on the flanks of the plateau are out of the scope of the study.

**Fig 14:**
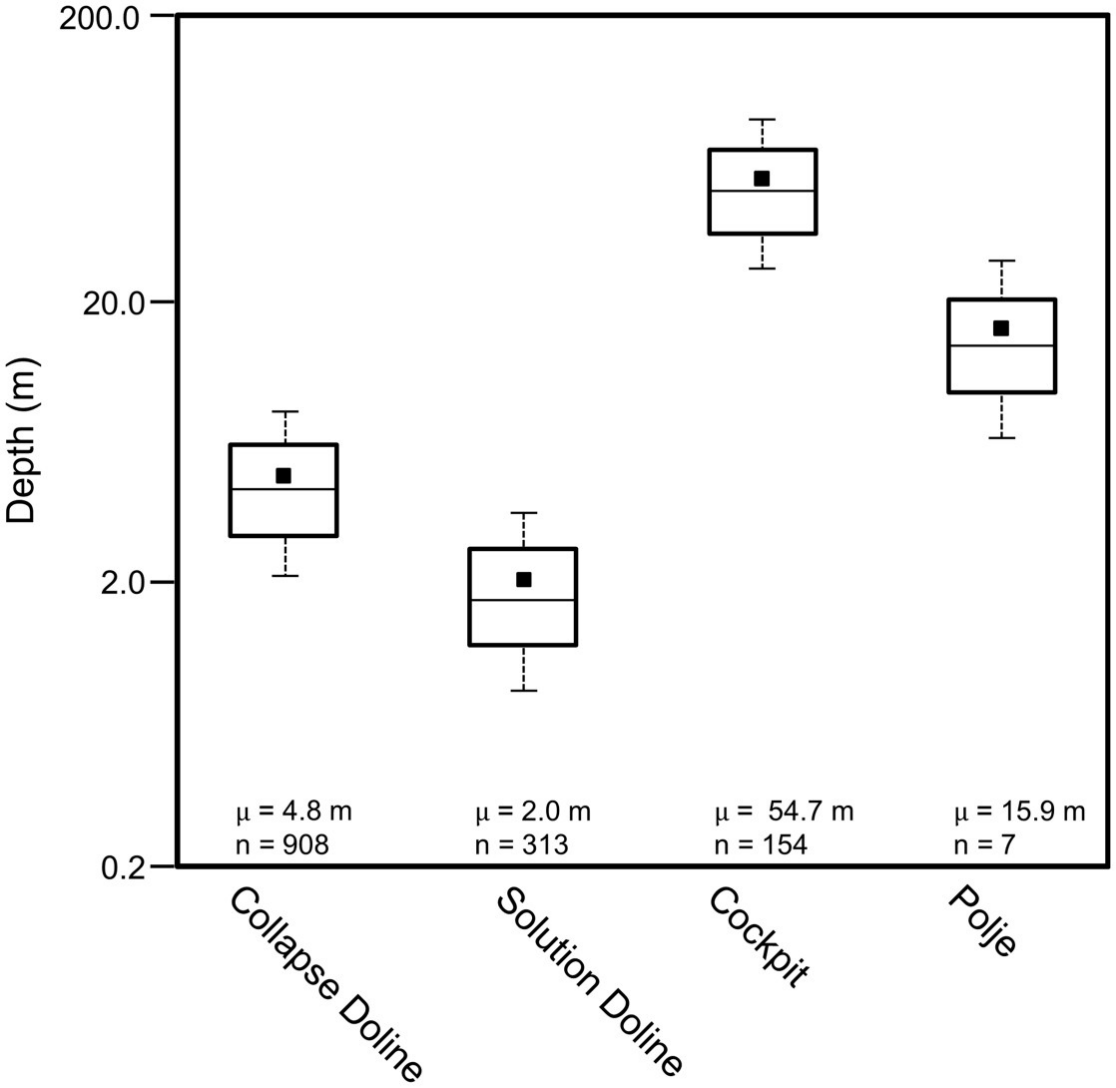
Box plots showing the depth of karst depressions. For collapse dolines and cockpits, the depth measured is the maximum depth, the difference in height between the maximum elevation of the perimeter and the lowest point of the depression [168]. Considering solution dolines and the poljes, the depth measured is the closed depression depth. For the small features, the P25/P75 distribution of collapse dolines is different from that of solution dolines. In a similar manner for large features, the P25/75 distribution for cockpits is different from that of poljes.

Dolines are among the most common macro-scale karst features in the MCKB as reported by numerous authors. Aguilar and others mapped dolines and other karst landforms in Yucatán showing that their distribution is related to the Chicxulub impact crater and other underlying structures [160]. Fragoso-Servón made a similar interpretation for the state of Quintana Roo and showed that the density of karst depressions was related to the underlying northeast trending structure of the RHFZ [161]. In a study of the karst geomorphology of the Calakmul Biosphere Reserve, Garcia and others mapped the distribution of dolines in the northern MCKB [162]. Dahlin reported a series of sinkholes on the flank of the La Muerta upland noting collapse features, large limestone blocks at the base, and quick drainage of water [152]. Since then, multiple authors have noted the presence of dolines [163–166] but they have not been studied extensively because they are difficult to map on satellite DEMs, which have low resolution and are adversely affected by the forest canopy. However, detailed interpretation of dolines on LiDAR data has shown distinct patterns. Collapse dolines, with a mean area of 263 m^2^, are typically present in chains along the flanks of the larger *bajo* depressions (Fig 12A). They have steep sides, small cliffs, show evidence of collapsed caves, and their bases are commonly strewn with collapsed rubble (Fig 12B). Faulting along the *bajo* margins created fracture systems that promote dissolution, cave formation and later collapse. These provide a focus for the collapse of the larger *bajos*. Solution dolines on the other hand are larger and scattered throughout the region. Commonly observed in the upland areas (Fig 12C), they have a mean area of 8249 m^2^ and typically measure a few meters in depth. Morphometric analysis of karst depressions (Figs 13 and 14) demonstrates a statistical separation between solution and collapse dolines.

#### Intermittent lakes

Intermittent lakes or ponds, varying seasonally or over a period of years or decades, are a pervasive phenomenon in karst basins [29,147,170]. Karst lakes may occur in upland areas associated with perched aquifers caused by the presence of thin marls or shales that are common within limestones [150]. Alternatively, vegetation debris or clay may occlude water flow into the subsurface conduit system in karst ponds with a high organic input [120,171,172]. Over time, the pond may come and go with erosion of the conduit plug, allowing the pond to drain and fill repeatedly. Intermittent lakes are commonly present in the upland areas of karst basins [173–175].

**Fig 15:**
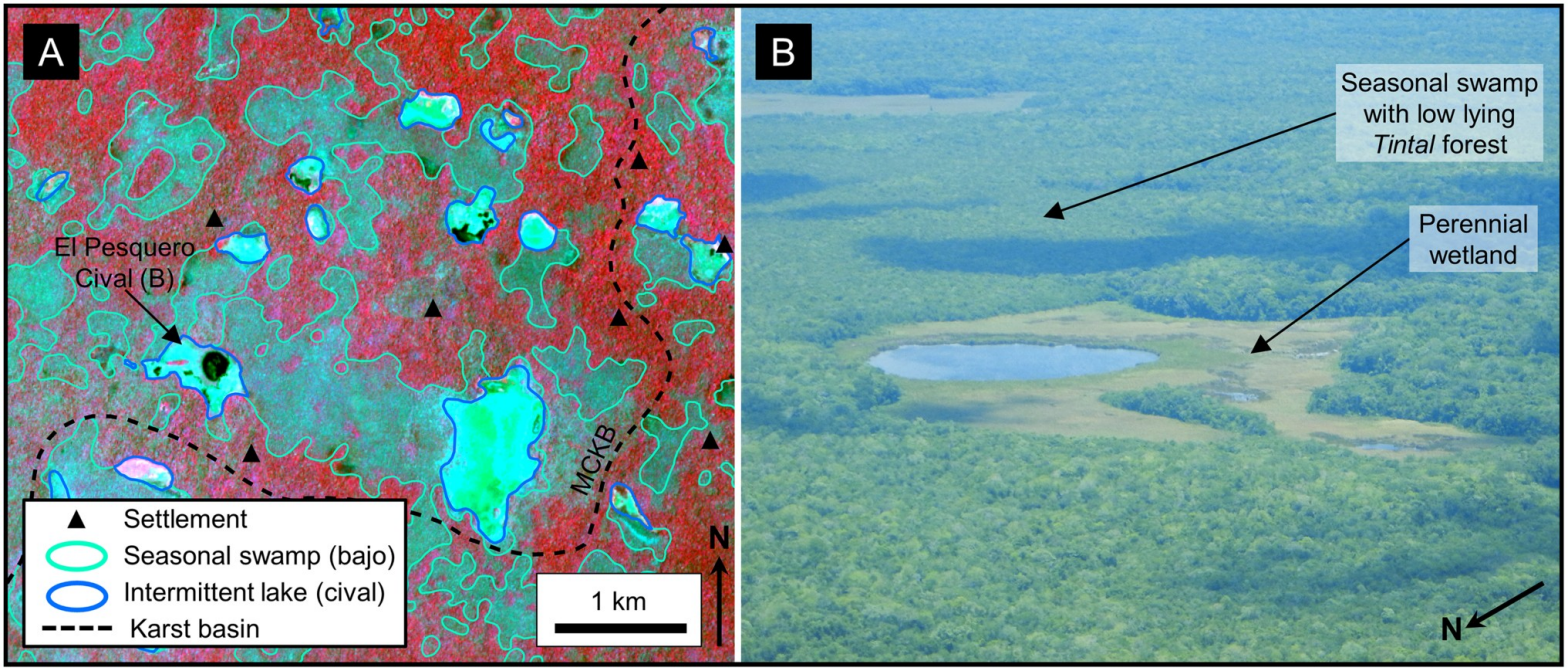
Intermittent lakes, or *civales*, along the southeast margin of the MCKB. (A) Sentinel-2 NIR image illustrates a cluster of intermittent lakes in an upland setting. The *civales*, characteristically represented by a cyan color, are perennial wetlands nested within the *bajos*. Open water, here seen as black, is present in some of the *civales* as small lakes or ponds. GoogleEarth historical images show that the water bodies have changed over the last 50 years, at times dry and at times wet. (B) Aerial photograph of the El Pesquero Cival, an example of an intermittent lake. Sentinel-2 multispectral data have been provided by CNES (https://cnes.fr/en) and printed under a CC BY 4.0 license.

Intermittent lakes, or *civales*, exist throughout the north Petén but are present in large numbers on the MA along the southeast side of the MCKB (Fig 15A and 15B). In describing the physiography of the YP, Wilson and Gates have both stated that the PP has numerous intermittent lakes [4,68]. Locally in Guatemala, intermittent lakes are commonly known as *civales* and are grassy wetlands that commonly have shallow bodies of water [176,177]. In the north central Petén, the water level of *civales* is non-perennial and has been known to fluctuate either seasonally [177] or over a period of years [136]. Within the MCKB specifically, the marshy *civales* usually stay wet throughout the dry season, but occasionally dry up on the surface [50]. Studies of the paleoecology of the upland Bejucal Cival, on the southeast flank of the PP, and the El Palmar Cival, in the lowlands immediately east of the Buenavista Escarpment, attributed the presence of standing water in karst depressions to clay plugging of the natural conduit system [176]. Multiple papers [34,48,85] describe *civales* in the MCKB. A joint project of the Instituto Geográfico Nacional with the Inter-American Geodetic Survey and the Defense Mapping Agency mapped *civales* along the MA as intermittent lakes [178,179].

#### Lack of streams

One characteristic of karst drainage basins is the lack of well-developed surface streams. In autogenic karst basins all precipitation sinks into the subsurface conduit system which is adequate under all flow conditions, and there is no surface water flow [29,180]. However, allogenic basins such as the MCKB maintain surface channels that flow intermittently [29]. During normal conditions water movement for such a basin is underground as surface flow seeps into the streambed through the alluvium or drains into subsurface conduits through swallow holes [157]. However, under exceptional flood conditions, surface flow exceeds the capacity of swallow holes or the conduit system and flows overland, downstream, and out of the basin [180–182]. Intermittent streams and swallow holes are common features of karst basins.

**Fig 16:**
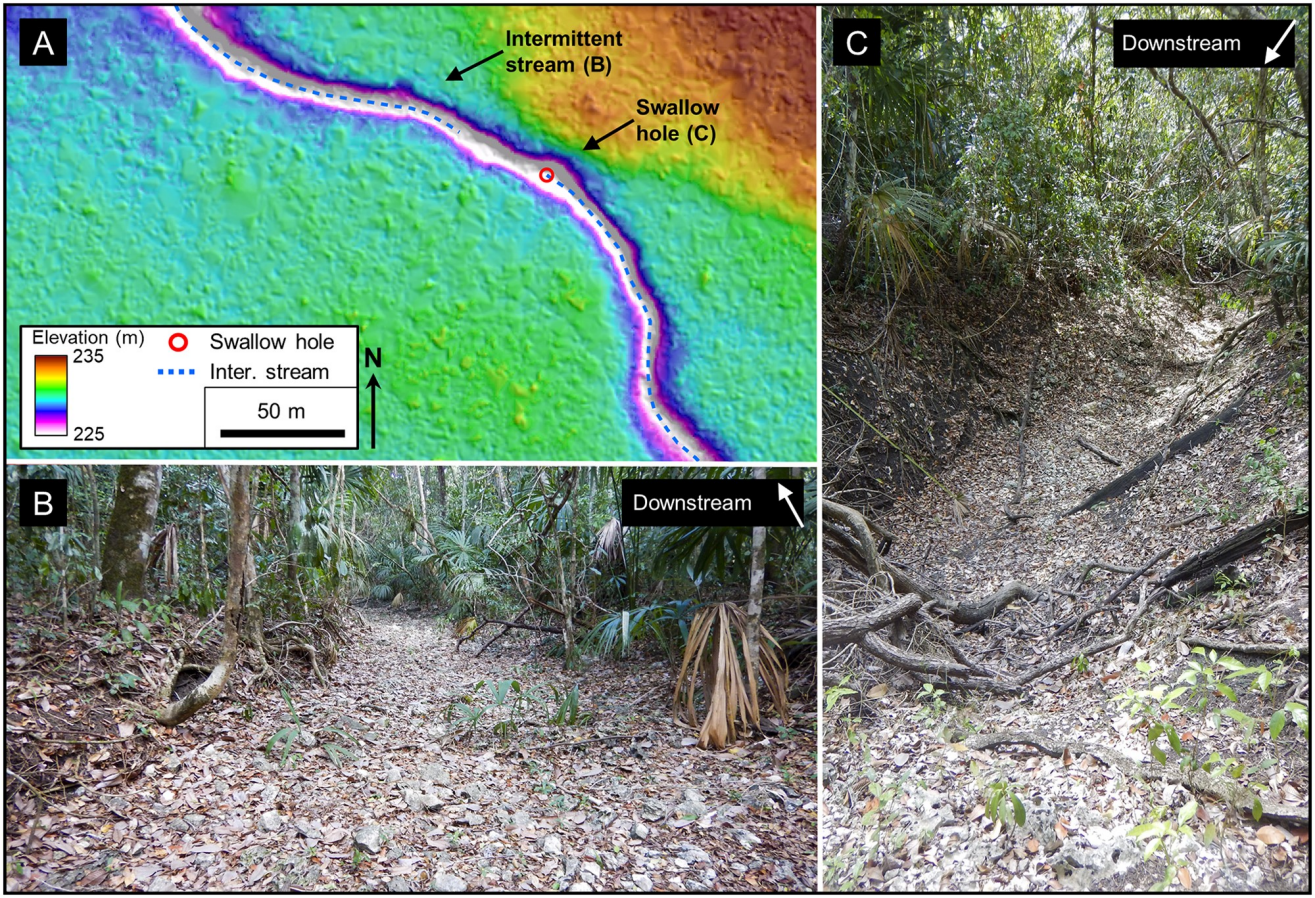
Intermittent stream and swallow hole. (A) LiDAR data showing the Arroyo Paixbancito north of the Los Pericos settlement. The streambed is 8–10 m wide and 1.5–2 m deep. (B) Arroyo Paixbancito at the end of the dry season showing a dry streambed covered by angular limestone cobbles. After seasonal storms, this arroyo carries overflow from La Jarrilla Bajo, west of El Mirador, into the half-blind Paixbancito Lagoon. In August 2013, the arroyo flooded with water greater than 2 m deep at this location, high enough for water to overflow the Paixbancito half-blind valley. (C) A swallow hole in the arroyo, 2 m deep, clogged by debris. In this section of the arroyo there is a similar swallow hole every 600 m on average. LiDAR data have been provided by FARES (https://www.fares-foundation.org/) and printed under a CC BY 4.0 license.

Intermittent streams are present throughout the irregular landscape of KVs of the MCKB and serve to drain surface water into the subsurface except in the case of large-scale floods where water overwhelms the conduit system. Geomorphologists have often described the southern PP as having a poorly developed surface drainage which feeds into a fracture network and conduit caverns [4,5,16]. Hydrologists studying the groundwater in the YP have stated that the region has no perennial rivers and drains only along ephemeral streams [43,183,184]. Gunn and others defined as intermittent two major arroyos that drain much of the northern MCKB [83]. Intermittent, or ephemeral, streams have been described and mapped over the course of several hyrogeological studies in the Mexican portion of the MCKB [39,55,162,185]. During early work in the Mirador region, authors reported the Río Seco, an intermittent stream in the La Jarrilla Bajo west of Mirador [152,153]. More recently, work by the Instituto Geográfico Nacional mapped intermittent streams in the southern MCKB based on satellite imagery [178,179]. We mapped using LiDAR data the channels of all intermittent streams present in the KVs and *bajos* (Fig 16A) and found them to have a mean length of 510 m (P90/P10 = 88/1206 m). Where observed in the field channels are commonly gravel lined (Fig 16B).

Drainage into the subsurface fracture system of the MCKB occurs through swallow holes and ponors. We identified two broad categories of swallow holes, both of which have a physical relief of 0.5 to 2 m. In the KVs, numerous swallow holes with small-scale depressions have broken the intermittent streams into short stretches (Fig 16C). Another setting in which swallow holes are common is the closed depressions associated with many KVs and large *bajos*. Often, they are present at the deeper end of the depressions along the faulted margins and tend to be broader depressions. This relationship is illustrated in a description of the *bajos* of northwest Petén made during a reconnaissance of the Candelaria River [117]. Depressions less than 0.5 m, where water seeps in the subsurface through alluvium, have been observed in the field but are not easily recognized on LiDAR data. Ponors, physical holes into the subsurface, that drain water into the subsurface, have only been noted in the field in a few instances in large solution pans north of Tintal.

#### Conduits

Conduits, or systems that transport water in the subsurface, may take the form of solution enhanced fractures and larger caves. Water that enters the subsurface in the epikarst, or shallow weathered limestone, travels through joints, fractures, bedding planes and solution features before reaching a more open conduit system [186]. Apertures for such pipe-like conduits may range in diameter from 1 cm to a few tens of meters [187]. In fluviokarst dominated basins, subsurface conduits underdrain each of the losing stream reaches [157] and coalesce into trunks that underdrain the larger KVs [121,188].

**Fig 17:**
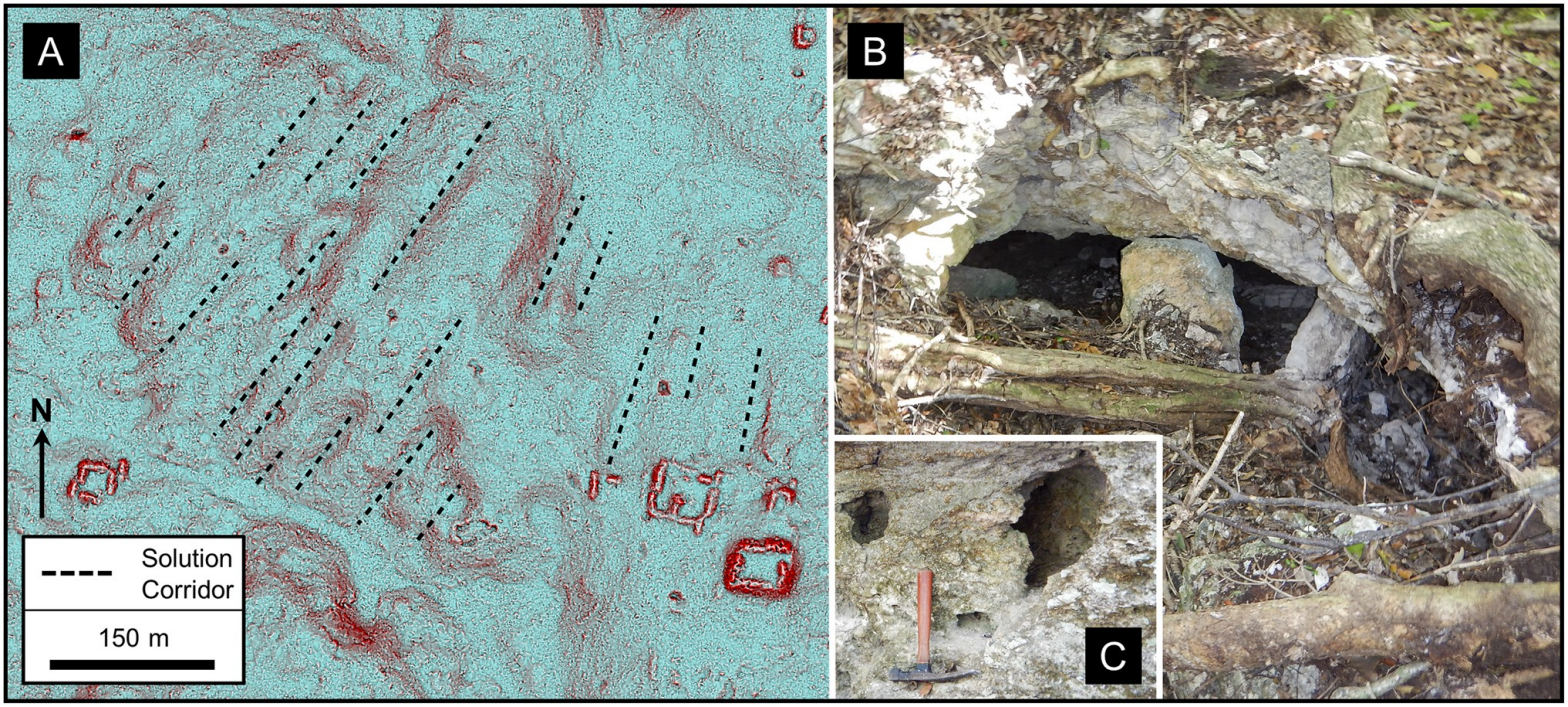
Surface evidence of the subsurface fracture system. (A) RRIM showing solution corridors northeast of the La Danta structure, karst features present in many parts of the southern MCKB. Measured fractures from outcrops in this area have an average strike of N44°E, close to the N35°E trend of faults in the RHFZ which bounds the Petén Plateau. We also map solution corridors on LiDAR data near Peñascal and Danto. (B) Collapsed entrance to a small cave, or solution pocket, near the Guacamaya Group in the El Mirador upland. Field teams have also observed caves and overhanging shelters associated with collapse dolines near Tintal, La Muerta, and La Danta. (C) Solution enhanced vadose tube seen in outcrop in the El Mirador region, with a rock hammer for scale. LiDAR data have been provided by FARES (https://www.fares-foundation.org/) and printed under a CC BY 4.0 license.

The subsurface conduit system of the MCKB comprises an extensive fracture network, minor caves, and vadose conduits in the near surface (Fig 17), all enhanced by dissolution. According to Bauer-Gottwein and others the preferential flow paths in the Yucatán include regional-scale fault zones, large-scale dissolution conduits, and small-scale fractures and dissolution cavities; all of which are present in the southern PP [43]. Although many karst basins drain through extensive conduit systems that include caves [11] karst basins within a fluviokarst terrain typically have only small caves and the KVs are underdrained by a solution enhanced fracture network. Although large caves are not known from within the MCKB they exist nearby. Šprajc identified caves on the eastern flank of the PP in southeastern Campeche [189,190]. The Grutas Colón, located just outside the northeastern edge of the MCKB, illustrates a variety of features typical of phreatic caves that drain karst basins [191]. Caves are present along the Buenavista Escarpment near El Zotz [192] and Uaxactun [193]. Numerous caves located in the cockpits of the El Zotz [194] serve to drain water from the cockpits into the subsurface. The extensional RHFZ, which has an overall strike of N35°E controls the fracture patterns in the southeastern Yucatán [195,196]. We confirmed the presence of this extensional regime in the MCKB through fieldwork that documented the interpretation of widespread solution corridors (Fig 17A), known in the Caribbean region as *zanjones*. Caves in the MCKB typically take the form of collapse features associated with dolines, solution pockets, and overhanging shelters (Fig 17B). In the southern MCKB vadose tunnels, pipe-like features in the undersaturated vadose zone above the water table, are present in large numbers (Fig 17C). This system of solution enhanced features present in the MCKB serves to funnel water into the subsurface conduit system that drains the basin.

#### Springs

Water that recharges the subsurface in a karst basin discharges through single or multiple springs that mark the downstream edge of the basin [28,150,180]. Underflow springs on the outer margins of the basin carry the base-flow of the basin. Such karst springs are the natural outlets for water discharging from the conduit network and, as such, represent the regional level of groundwater [11]. Overflow springs in the interior of the basin discharge during spillover conditions of the base-level conduit system. When perched water tables are present in the basin ephemeral springs may flow at a point where the aquitard outcrops locally [28,44]. Spring flow can be perennial with a degree of variability over the course of a year [197], flow may appear only temporarily when they are charged by a yearly rainy season, or discharge may be activated only after a particular precipitation event following several years of inactivity [198].

**Fig 18:**
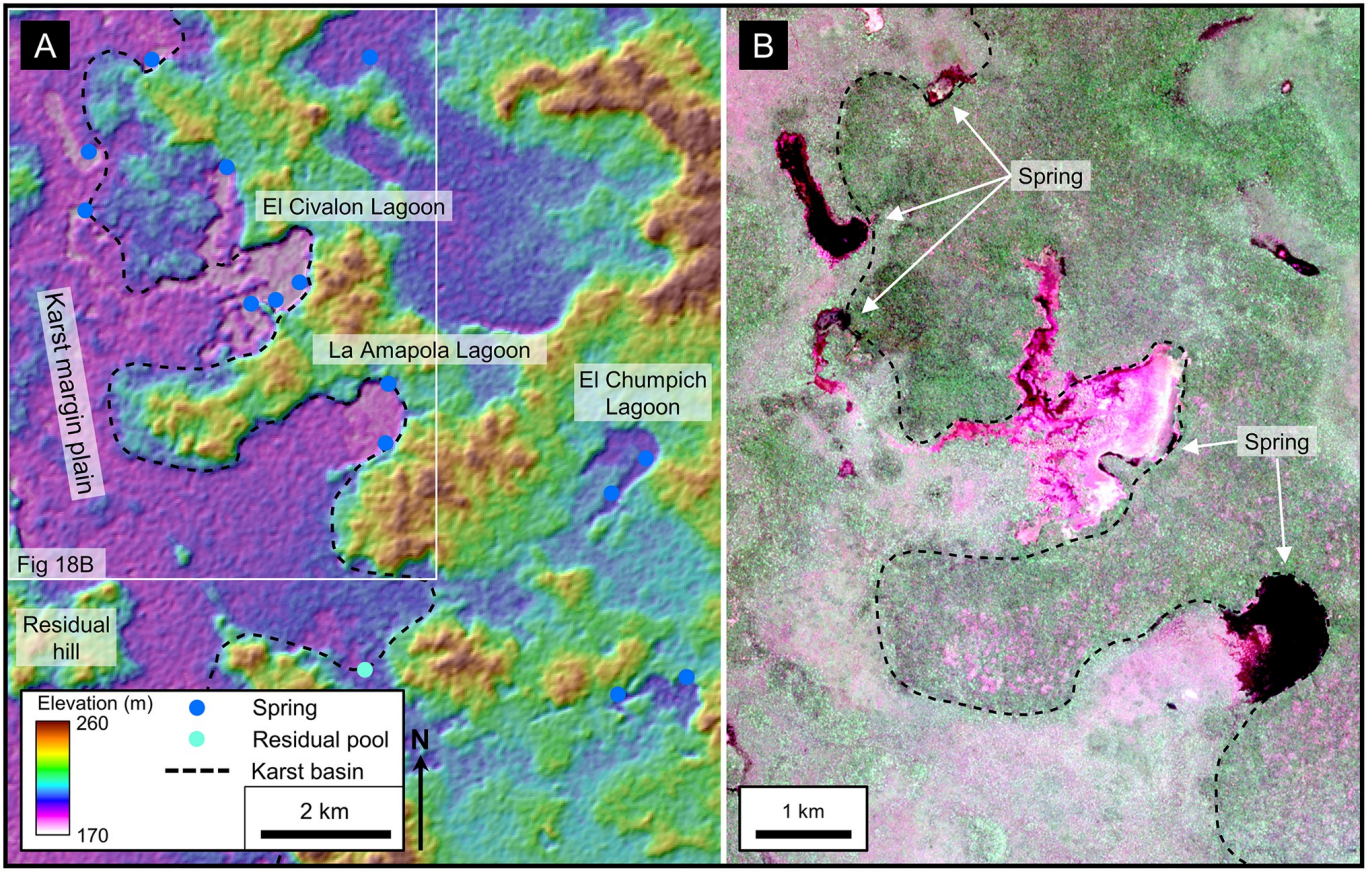
Steephead valleys and exsurgence springs along the west margin of the MCKB. (A) AW3D30 DEM from the western margin of the MCKB showing the typical topography along the basin margin composed of a chain of low hills punctuated by occasional dry valleys. Most notable are the exsurgence springs at the base of the sharp slope identified using satellite data and aerial photographs, with a smaller number of springs located in the interior of the basin. Two steephead valleys are present headed by El Civalon and La Amapola lagoons, 2.5–3.5 km long with prominent walls 30–60 m high. Each of the valleys has multiple springs which have contributed to the valley growth through spring sapping creating an amphitheater-like lagoon. One steephead, El Chumpich Lagoon, is present in the interior of the basin. In the southern part of this region a residual pool is located where a karst valley, and its intermittent stream, exits the basin. Drainage along this overflow channel flows towards Arroyo Cuba to the west. A residual hill in the karst margin plain is present in the southwest. It is this region between the wetlands to the west and the seasonally dry uplands to the east that Scholes and Roys [205] referred to as the lacustrine belt. (B) Sentinel-2 TCI data from mid-June 2020 showing the La Amapola Lagoon and other springs filled with water following a storm event. Given that there is little surface catchment area associated with the lagoon we assume the springs sourced the water. AW3D30 elevation data have been provided by JAXA (https://www.eorc.jaxa.jp/ALOS/en/aw3d30/) and printed under a CC BY 4.0 license. Sentinel-2 multispectral data have been provided by CNES (https://cnes.fr/en) and printed under a CC BY 4.0 license.

Springs and seeps are present along the western margin of the MCKB and within the interior (Fig 18). Previous authors have noted the presence of springs in the upland areas of the PP and the interior of the basin, although not plentiful in number [16,68,199,200]. On the flanks of the plateau, springs have been identified on the southeast at El Manantial [201] and El Palmar [135,136], in the south draining to the San Pedro River [202], and in the west draining into the Candelaria and Julubal rivers [117]. Underflow springs along the west flank of the basin include small seeps and larger complexes of distributary springs. Steephead valleys, short valleys terminating in an amphitheater at the head [120], are present along the western flank of the MCKB and in the interior (Fig 18A and 18B). Although most springs flow during and after the rainy season, some appear to flow during the dry season as well [203]. Over time, spring sapping has created the line of low hills that mark the western edge of the basin. Without field verification, we argue that interior springs previously seen by field parties [204] and those identified from remote sensing data by us are ephemeral overflow springs.

#### Basin components

In well-developed karst drainage from distant recharge areas collect groundwater from intervening subsurface tributaries into master trunk flow [29]. Like surface streams and valleys, the subsurface drainage network is usually dendritic with tributaries coalescing into master trunks [181,188]. Hydrogeologists routinely document karst drainage through dry tracer tests which document the flow path of groundwater from a swallow hole to a spring. However, most basins lack demonstrable intrabasin flow paths and hydrogeologists infer flow paths without implying exact locations [29]. They make such inference knowing that the underground drainage pattern typically matches the surface stream system [120].

**Fig 19:**
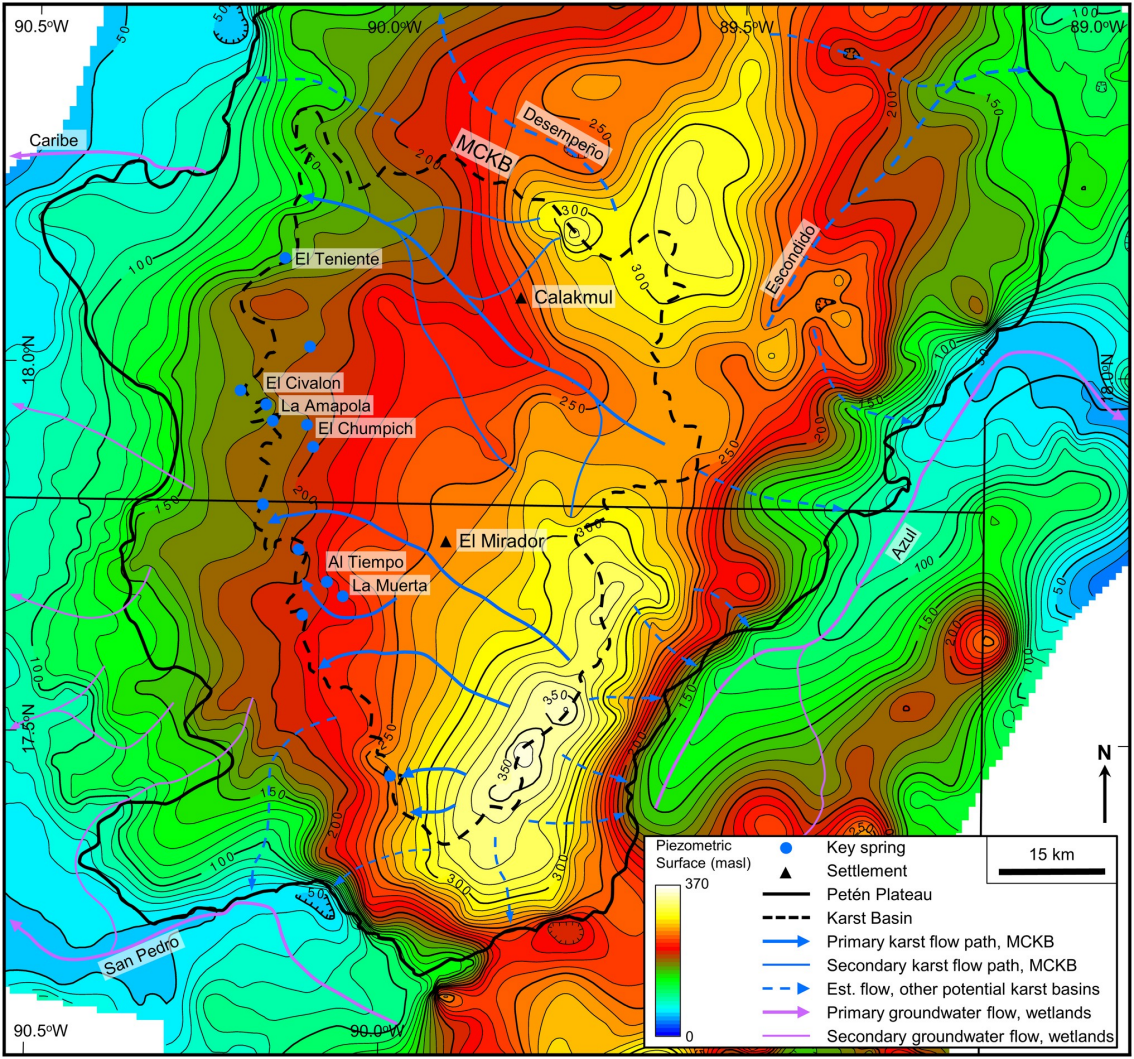
Regional groundwater map of the southern YP showing subsurface flow paths for the MCKB. We captured a dataset of over 4000 hydrologic features, listed in Table 3, which included observation times covering multiple seasons. These were gridded in the Kingdom software using a grid interval of 1000 m and displayed the resulting piezometric surface with a 10 m contour interval. Given variations in hydrogeologic conditions over different observation times, we believe that this mapped surface is regional in nature. We show interpreted flow paths in the karst basin and the surrounding area with names of the associated rivers, with flow interpreted normal to the piezometric contours. Outside of the MCKB on the Petén Plateau, we further show flow paths for potential karst basins that border the MCKB. In karst basins marginal springs occur where the regional groundwater surface intersects the topography, typically at a break in the slope or a stratigraphic or fault contact where less permeable rocks restrict conduit development [28]. The western boundary of the MCKB and the adjoining karst margin plain is a locus for springs given the dramatic slope break and the observed intersection of the topography and the mapped piezometric surface. All layers were produced by the authors and are copyright-free.

We infer subsurface flow paths in the MCKB to have a dendritic pattern of trunk conduits and tributaries that underdrain the KVs and flow to the west and northwest. The central Yucatán anticlines control the primary direction of groundwater flow in the PP and deflect water either to the east or the west [16,68]. More recent research in the YP has shown the dynamic relationship of groundwater movement and cave systems [41]. A conceptual model for eastern Campeche and Quintana Roo shows recharge in the upland *bajos* and eastward subsurface flow in cave systems [39]. Also, Bauer-Gottwein and others reported that groundwater modeling by multiple groups demonstrates a bidirectional flow pattern in the Mexican portion of the YP [43]. They later extended the modeled area to include both Mexico and northern Guatemala. Their results demonstrate groundwater flows west and east from the MA and CA and, in the MCKB specifically, flow paths trending west northwest. Given the absence of dye tracer data we modeled the regional groundwater for the southern YP integrating over 4000 natural water points as outlined above. Fig 19 shows a regional groundwater map modeled from these data and flow paths for the MCKB interpreted to follow the troughs in the piezometric surface [28]. Water flows in the subsurface from the uplands on the east and northeast of the basin toward the west margin, following the troughs of the water table, with a dendritic pattern that mirrors the major KVs.

Hydrogeologists delineate karst basin boundaries based on a variety of information including geologic structure, topography, and groundwater data in the form of groundwater maps or dye tracer tests [197]. In its simplest form, the upstream limits of a subsurface groundwater basin and its related surface watershed are identical and can be mapped from topographic divides, an accepted practice when tracer data are lacking [8,29,44,46,159]. In more complex cases, groundwater may move underneath surface watersheds divides. In both cases, karst springs define the downstream margin of the basin [8,28]. Karst basins may be singular or may include multiple sub-basins [28,46,158,206].

The boundaries of the MCKB are the crest of the Mirador Anticline on the southeast, the crest of the Calakmul Anticline on the northeast, the drainage limit of the Calakmul Arroyo on the north, and the line of low hills on the west marked by springs (S3 File). The CA controls both the surface and the subsurface drainage in the Yucatán with groundwater moving from the crest to both the east and the west [16,43,63]. Given the lack of tracer data in the southern PP, we honored topographic divides to map the northern and eastern basin boundaries. This is consistent with studies that document most karst aquifer drainage coincides with topographic watersheds [29,170]. On the north side of the basin, the divide between the Desempeño and Calakmul drainages is a line of hills trending northwest. As recognized by Gunn and others, the CA, truncated by the eastern end of the El Laberinto Bajo, is the eastern limit of the Calakmul area [21]. The MA, which rises from its intersection with the El Laberinto Bajo and trends south-southwest, controls the drainage for the southern MCKB [37]. These northern and eastern limits for the Mirador and Calakmul watersheds are consistent with drainage analyses by the USGS [112,113,207] and the hydrological surveys of Guatemala and Mexico [105,109,111,185]. On the western side of the basin, we interpret that the boundary of the MCKB is the low line of hills 20–40 m high that marks the eastward erosion limits of the karst margin plain. Within the MCKB, the Mirador Sub-Basin and the Calakmul Sub-Basin have been separately mapped, also based on their respective surface watersheds [28,29,197]. Groundwater recharge in the interior of the basin emerges as a series of seeps, karst springs, spring-fed lagoons, and steephead valleys located along these hills.

We classify the MCKB as an overflow allogenic karst drainage basin where subsurface piracy of surface streams recharges the subsurface. Such basins tend to be larger and more immature. The hydraulic capacity of the conduit system in these basins has developed to manage the base or moderate flow conditions, but intermittent or storm-overflow routes maintain surface channels [11,29,158]. This is the case of the MCKB where the intermittent streams are composed of short segments interspersed with swallow holes. In storm events water flows beyond the swallow holes and down the KVs, or ponds in the closed depressions of the *bajo* system. Other basin types are moderate-sized underflow allogenic basin where all surface flow is diverted underground and the KVs become blind, and small-sized local autogenic basins where all surface flow is captured through direct recharge from the land surface with internal runoff into dolines.

A drainage map showing karst hydrologic features, boundaries, and inferred flow paths is the accepted manner to document results of a hydrogeologic study of a karst basin. Particularly notable amongst published maps is a high-quality atlas that documents numerous karst basins in the Kentucky Pennyroyal and Inner Bluegrass karst terrains with the publication of 13 drainage maps [173,174,175,206]. Karst hydrology of the Valley and Ridge Province of West Virginia has been documented through publications which portray the karst basins of Greenbriar and Monroe counties, each with multiple sub-basins [197]. Other notable examples of karst drainage basin maps include the Mitchell Plateau of Indiana [121], the Devil’s Icebox Basin of central Missouri [208], multiple basins in the Devonian limestone of New York [209]. Karst basins documented internationally include China [210], France [211], Mexico [212], and Puerto Rico [213].

A drainage map of the Mirador-Calakmul Karst Basin shows the key hydrologic features of the basin (Fig 10 and S2 Map). We constructed the MCKB drainage map following the style symbology commonly used for drainage maps by the US Geological Survey and others [11,29,30,182,206].

## Conclusion

Our work synthesizes geological, geomorphological, and hydrological data to define the Mirador-Calakmul Karst Basin of the Petén Plateau. We use these data to summarize the structural history of the PP from Paleocene to present, map the distribution of large-scale karst landforms and small-scale karst hydrologic features, classify karst landscapes, validate the presence of the Mirador-Calakmul Karst Basin, and model groundwater flow.

This paper underlines the importance of applied GIS-based mapping to the study of geomorphology in karst regions. As a result, we classify the southern PP into five karst landscapes, each with a dominant karst landform:

1. Fluviokarst, a landscape in which the geomorphology is transitional from fluvial erosion to karst dissolution, covers a significant portion of the southern PP. Karst valleys occupied by seasonal wetlands are the dominant landform.
2. Polygonal karst, a rugged cockpit and cone terrain dissected by dry valleys, is present along the southeastern margin of the plateau. The cockpits have large numbers of caves, collapse structures, and swallow holes that drain water quickly into the subsurface.
3. A karst margin plain lies on the southwestern flank of the plateau. Residual hills, remnants of an older fluviokarst region, lie on an alluvial plain pockmarked by wetlands. Steepheads mark the eastern boundary of the plain where spring sapping erodes into the fluviokarst.
4. Upland karst, a landscape with numerous intermittent lakes and grassy wetlands, is present along the crest of the Mirador Anticline.
5. Polje karst at the northern end of the plunging Calakmul Anticline has scattered baselevel poljes, many with small wetlands, indicating control of the regional water table.

We map key karst hydrological features that define the MCKB through integration of multiple forms of remote sensing data including orthophotographs, a satellite DEM, satellite TCI and NIR images, and a LiDAR survey as follows:

1. Seasonal swamps, recognized locally as *bajos*, are present in karst valleys, poljes, and solution dolines. Closed depressions are common in the karst valleys, poljes, and dolines. En echelon faults rim many of the karst valley depressions and the poljes.
2. Dolines are present in large numbers in the basin. Collapse dolines rim *bajos* indicating dissolution along deeper fault zones. Solution dolines present throughout the uplands.
3. Perennial streams do not exist in the basin. Instead, intermittent streams are present in the karst valleys. Swallow holes are present along the margins of *bajo* depressions, dolines, and interspersed along the length of stream channels.
4. Intermittent lakes and associated perennial wetlands are present in the upland area along the Mirador Anticline and in several *bajos* of the area. These ephemeral hydrological features lie within karst depressions and trap water by clogging of the conduit system.
5. Solution corridors, caves, and vadose tubes provide surface evidence of the subsurface solution-enhanced conduit system. In this send, solution corridors indicate the extent of fracture enhancement. Collapsed caves and vadose tunnels show the degree of subsurface dissolution.
6. Numerous steephead valleys, springs, and seeps are present at the base of a line of hills on the western side of the basin.

A drainage map depicts these karst hydrologic features along with inferred subsurface flow paths and boundaries of the karst basin. We argue that integration of surface water features, along with topographical data, demonstrates westward groundwater flow within the basin. We infer subsurface flow paths that underdrain the major karst valleys and represent these in a dendritic pattern. We further delimit the upstream boundaries of the MCKB based on topographic divides between watersheds in the absence of groundwater tracer data, in part following the crest of the Mirador and Calakmul anticlines. A line of springs delineates the downstream boundary of the karst basin at the base of a string of low hills punctuated by dry karst valleys.

During the dry season, when the water table is low, water in the basin is scarce but is still present in the *civales* and in residual lagoons along the western margin. When the rainy season starts, thousands of dolines and swallow holes that dot the landscape funnel water into the subsurface. Solution enhanced fractures and conduits in the limestone channel groundwater westward where it resurfaces at numerous springs at the base of the low hills along the basin’s west margin. Occasionally, heavy rains overwhelm the capacity of the conduit system and water flows on the surface in numerous channels and arroyos in the KVs. During these flood events the water table is high and overflow springs in the basin may be active as well. Much of the surface water ponds in the enclosed depressions and half-blind valleys scattered throughout the *bajo* system, but some eventually flows out of the basin margin.

**Fig 20:**
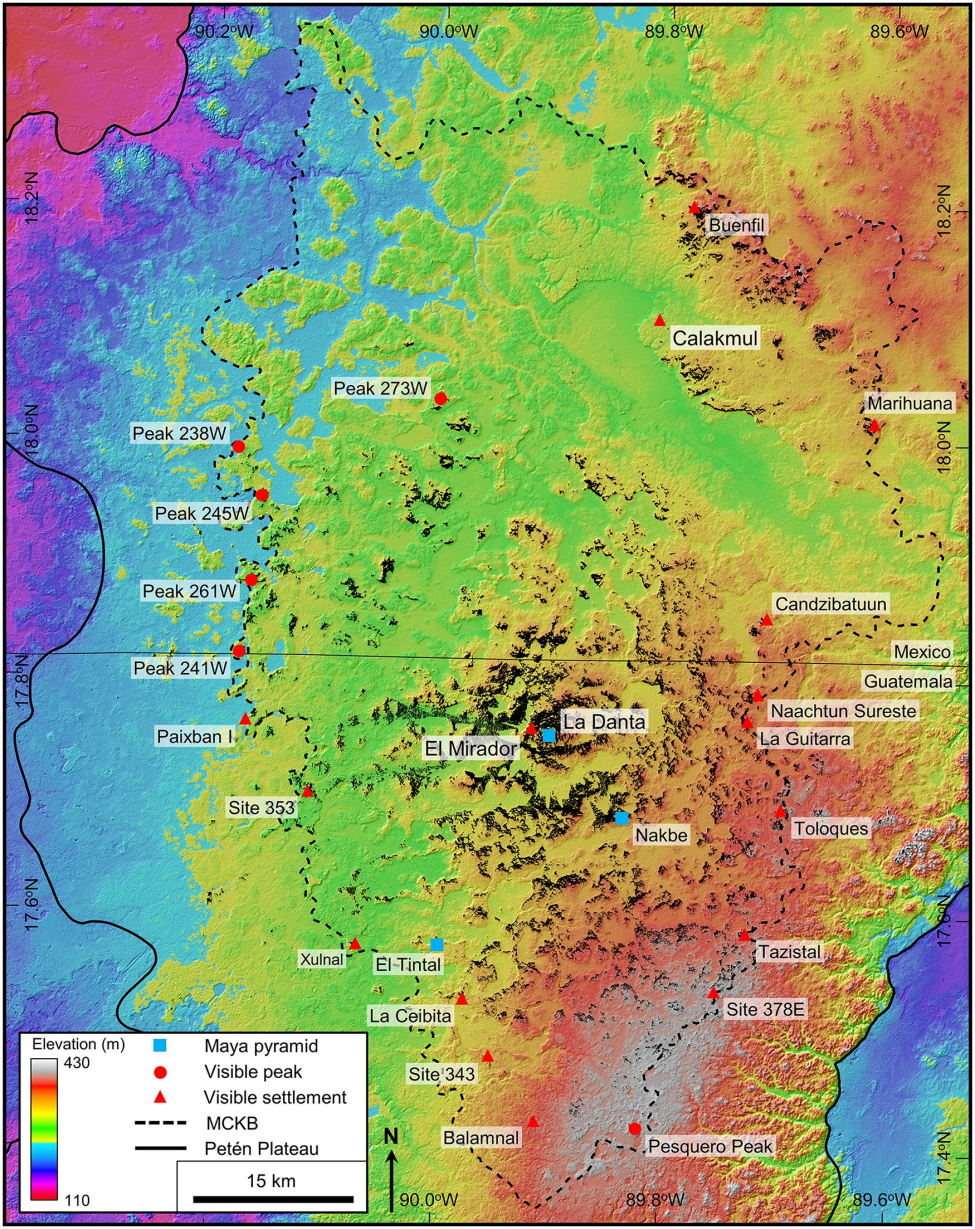
Viewshed map of the MCKB. Topography of the southern Petén Plateau, represented with the AW3D30 DEM, shows the surface configuration of the region. Both the MA in the south and the CA in the north are visible as broad topographic highs related to structural anticlines in the subsurface. The MCKB is outlined as previously defined. A viewshed map, shown by the black overlay, highlights the area visible from the top of the La Danta pyramid. We processed this overlay in ArcGIS using the Viewshed2 Geoprocessing Tool. Parameters used included an observer point at a height of 1.5 m, an outer radius of 60 km, and a refractivity coefficient of 0.13. We selected the Viewshed2 Tool over other options to account for the curvature of the earth. From the top of La Danta pyramid it is possible to see upland areas over most of the MCKB as follows: (1) To the north, a line of hills 32 km long from just west of Buenfil to the site of Marihuana on the northeast, (2) on the east flank, settlements along crest of the MA from Candzibatuun at the north end, through Naachtun Sureste and La Guitarra, and as far south as Pesquero Peak, (3) on the southwest margin, settlements including La Ceibita, Balamnal, Xulnal, and Paixban, and (4) along the west side, a broken line of hills from Peak 241W to Peak 238W in a region were sites have not been mapped. Beyond Peak 273W to the northwest, 135 km^2^ of the basin are not visible from la Danta. A large format version of this map is available online as Supporting Information (S3 Map). AW3D30 elevation data have been provided by JAXA (https://www.eorc.jaxa.jp/ALOS/en/aw3d30/) and printed under a CC BY 4.0 license. All other layers were produced by the authors and are copyright-free.

The Mirador-Calakmul region has a distinctive combination of uplands and lowlands which are unlike the surrounding landscapes dominated by other karst landforms. To the east a rugged polygonal karst terrain offers little in the way of *bajos* with soil for creating agricultural terraces. To the west is a mature karst margin plain with minimal upland areas for agriculture. The fluviokarst landscape offered the right mix of lowlands and uplands that enabled the Maya to develop their Preclassic civilization and thrive for several millennia. In sociopolitical terms, the ancient Maya of El Mirador would have seen a large portion of this exceptional karst landscape as illustrated by a viewshed map (Fig 20 and S3 Map). This map represents the features visible from La Danta, the most impressive pyramid in El Mirador and the highest point as well. From their city in the center of the basin the Maya observed their territory and several massive pyramids in the other major cities of Calakmul, Tintal, and Nakbe along with a host of other lesser sites. During the apogee of El Mirador (ca. 300 BCE), the Maya could view the extent of the karst landscape from the hills along the Desempeño divide, to the Mirador and Calakmul anticlines, and portions of the punctuated western ridge. Our GIS-based approach, integrating several forms of remote sensing data, has enabled us to interpret landscapes across the Petén Plateau, detect hydrogeologic features, and produce a drainage map of the Mirador-Calakmul Karst Basin.

## Supporting information

S1 Map Near-Infrared image of the Mirador-Calakmul Karst Basin. Sentinel-2 multispectral data have been provided by CNES (https://cnes.fr/en) and printed under a CC BY 4.0 license. AW3D30 elevation data have been provided by JAXA (https://www.eorc.jaxa.jp/ALOS/en/aw3d30/) and printed under a CC BY 4.0 license.
(PDF)

S2 Map Drainage map of the Mirador-Calakmul Karst Basin. AW3D30 elevation data have been provided by JAXA (https://www.eorc.jaxa.jp/ALOS/en/aw3d30/) and printed under a CC BY 4.0 license. All other layers were produced by the authors and are copyright-free.
(PDF)

S3 Map Viewshed map of the Mirador-Calakmul Karst Basin. AW3D30 elevation data have been provided by JAXA (https://www.eorc.jaxa.jp/ALOS/en/aw3d30/) and printed under a CC BY 4.0 license. LiDAR data have been provided by FARES (https://www.fares-foundation.org/) and printed under a CC BY 4.0 license. All other layers were produced by the authors and are copyright-free.
(PDF)

S1 File KML file of the Petén Plateau. All layers were produced by the authors and are copyright-free.
(KML)

S2 File KML file of the karst landscapes of the Petén Plateau. All layers were produced by the authors and are copyright-free.
(KML)

S3 File KML file of the Mirador-Calakmul Karst Basin. All layers were produced by the authors and are copyright-free.
(KML)
