# Supplementary material for: Geomorphology of the Mirador-Calakmul Karst Basin: A GIS-based approach to hydrogeologic mapping
Source: PLoS One. 2021 Aug 2;16(8):e0255496. doi: 10.1371/journal.pone.0255496 (PMC8328328; doi:10.1371/journal.pone.0255496)

Near-Infrared Image, Mirador-Calakmul Karst Basin

Ross Ensley, Richard D. Hansen, Carlos Morales-Aguilar, and Josie Thompson

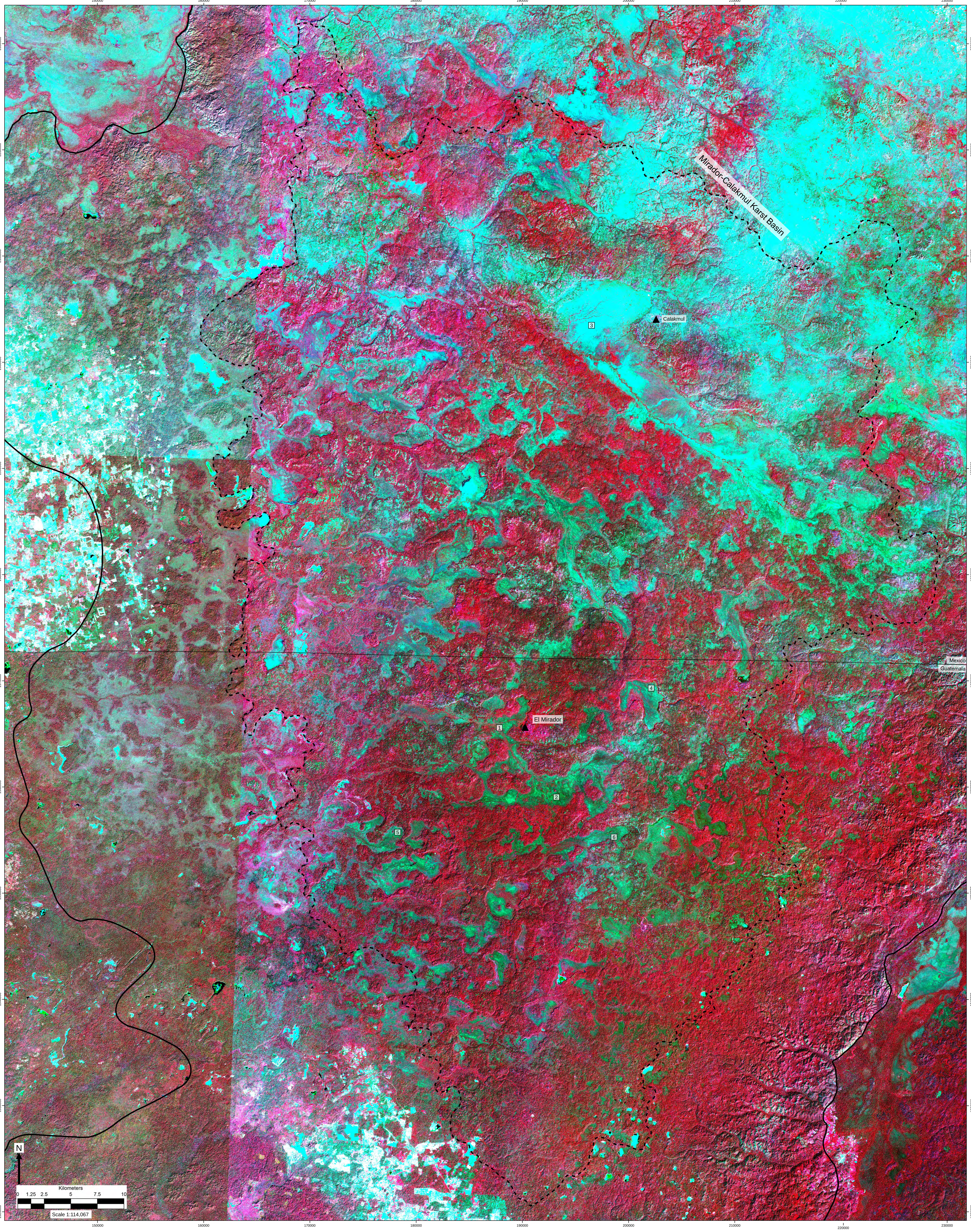

Legend
- Petén Plateau
- Karst basin
- Settlement
- Spring
- 1 TCI Bajo images

Map Projection: UTM Zone 16N
Datum: WGS 1984
Spheroid: WGS84

Sentinel-2 multi-spectral data, including near-infrared bands, were provided by the European Space Agency and the National Centre for Space Studies (CNES)

Sentinel-2 True Color Images (TCI) of bajos

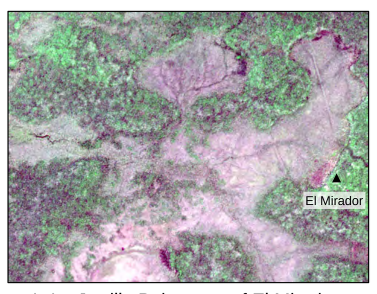

1. La Jarrilla Bajo, west of El Mirador

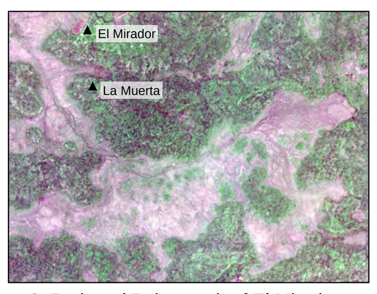

2. Pedernal Bajo, south of El Mirador

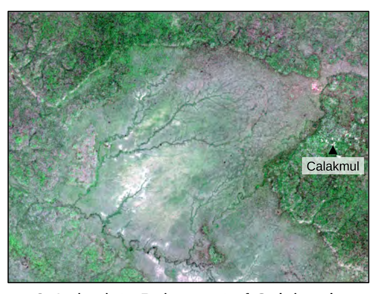

3. Laberinto Bajo, west of Calakmul

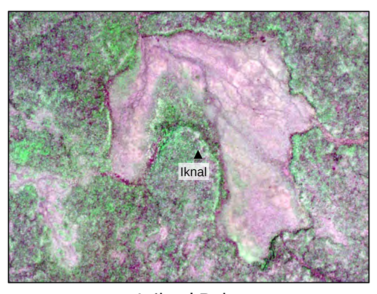

4. Iknal Bajo

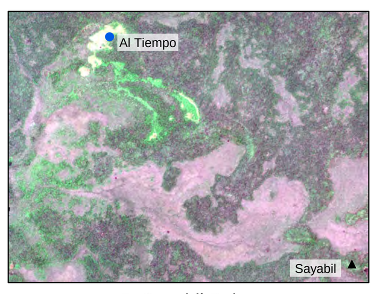

5. Sayabil Bajo

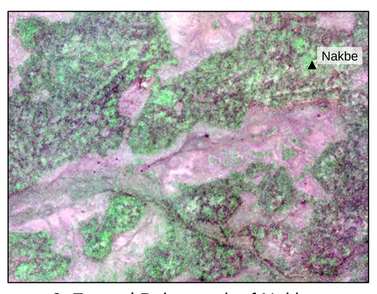

6. Zacatal Bajo, south of Nakbe

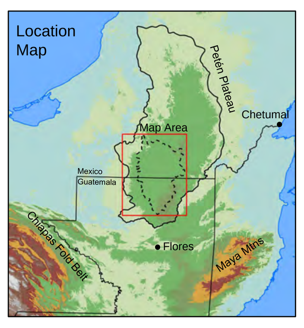

Supplement: S1 Map — Sentinel-2 multispectral data have been provided by CNES (https://cnes.fr/en) and printed under a CC BY 4.0 license. AW3D30 elevation data have been provided by JAXA (https://www.eorc.jaxa.jp/ALOS/en/aw3d30/) and printed under a CC BY 4.0 license. (PDF) [file pone.0255496.s001.pdf]
