# Supplementary material for: Geomorphology of the Mirador-Calakmul Karst Basin: A GIS-based approach to hydrogeologic mapping
Source: PLoS One. 2021 Aug 2;16(8):e0255496. doi: 10.1371/journal.pone.0255496 (PMC8328328; doi:10.1371/journal.pone.0255496)

Drainage Map, Mirador-Calakmul Karst Basin

Ross Ensley, Richard D. Hansen, Carlos Morales-Aguilar, and Josie Thompson

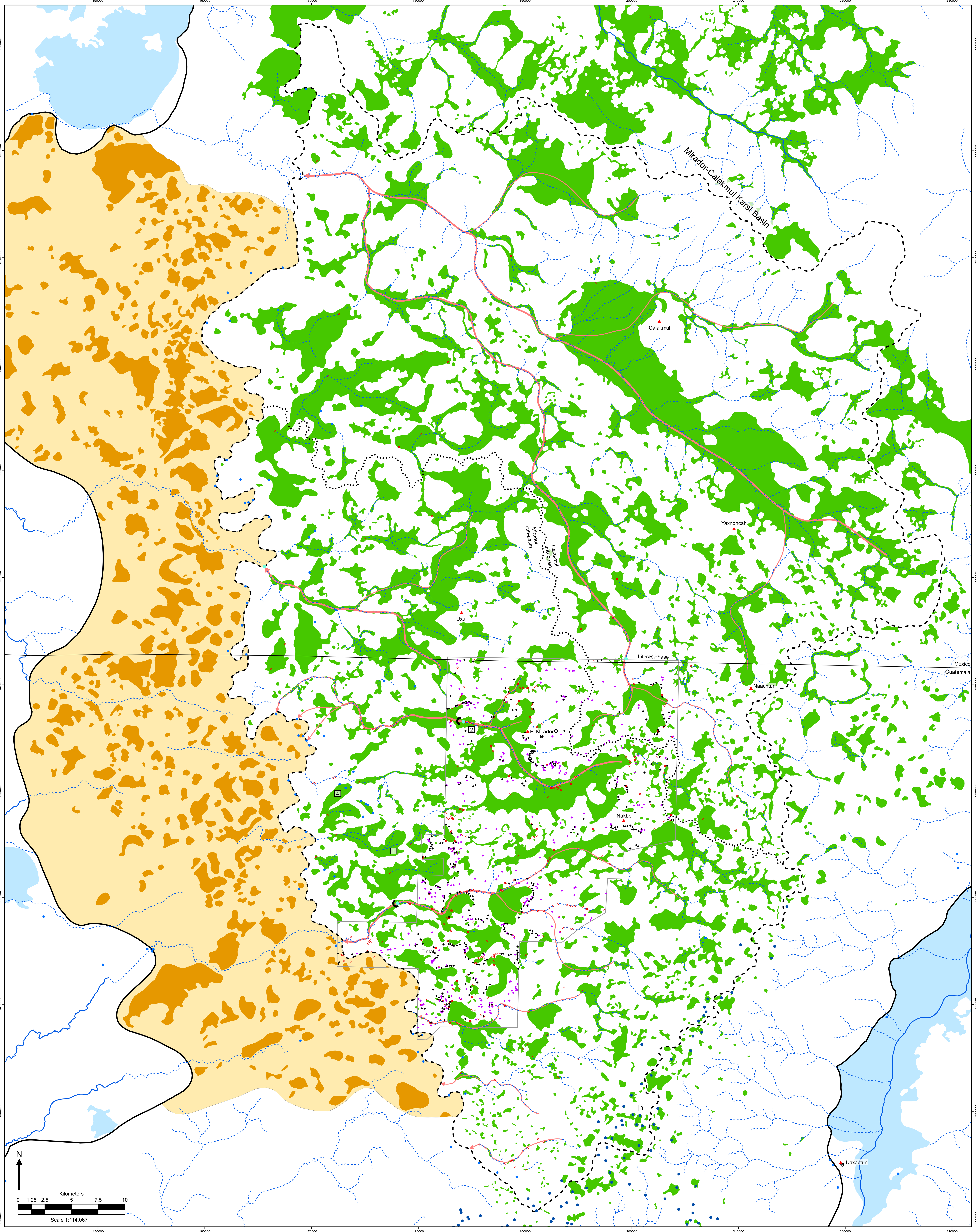

Legend

- Settlement
- Cave
- Swallow hole
- Spring
- Residual pool
- Intermittent lake (cival)
- Half-blind valley
- Collapse doline
- Solution doline
- Photograph
- Petén Plateau
- Karst basin
- Karst sub-basin
- River
- Intermittent stream
- Inferred subsurface flow path
- Phase I LiDAR survey
- Seasonal swamp (bajo)
- Residual hill
- Karst margin plain
- Wetland

Map Projection: UTM Zone 16N
Datum: WGS 1984
Spheroid: WGS84

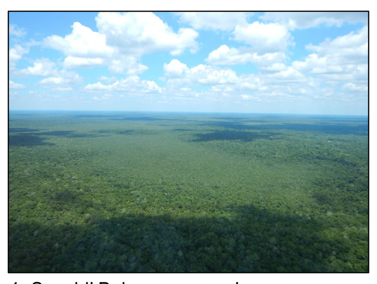
1. Sayabil Bajo, a seasonal swamp on the west side of the karst basin.

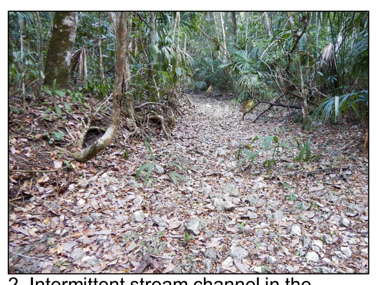
2. Intermittent stream channel in the Paixbancito Arroyo.

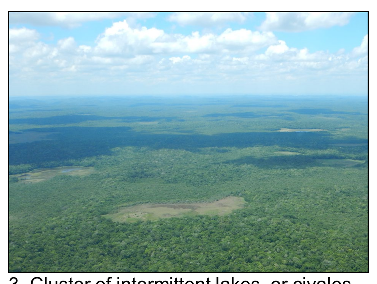
3. Cluster of intermittent lakes, or civales, along the southeast margin of the basin.

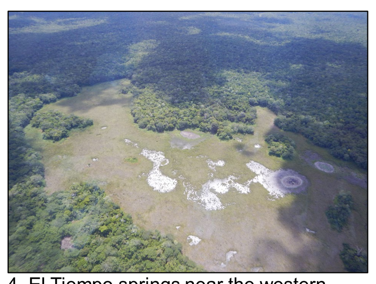
4. El Tiempo springs near the western edge of the karst basin.

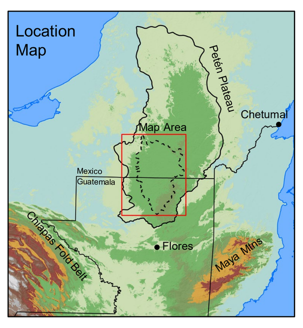

Supplement: S2 Map — AW3D30 elevation data have been provided by JAXA (https://www.eorc.jaxa.jp/ALOS/en/aw3d30/) and printed under a CC BY 4.0 license. All other layers were produced by the authors and are copyright-free. (PDF) [file pone.0255496.s002.pdf]
