# Supplementary material for: Geomorphology of the Mirador-Calakmul Karst Basin: A GIS-based approach to hydrogeologic mapping
Source: PLoS One. 2021 Aug 2;16(8):e0255496. doi: 10.1371/journal.pone.0255496 (PMC8328328; doi:10.1371/journal.pone.0255496)

Viewshed Map, Mirador-Calakmul Karst Basin

Ross Ensley, Richard D. Hansen, Carlos Morales-Aguilar, and Josie Thompson

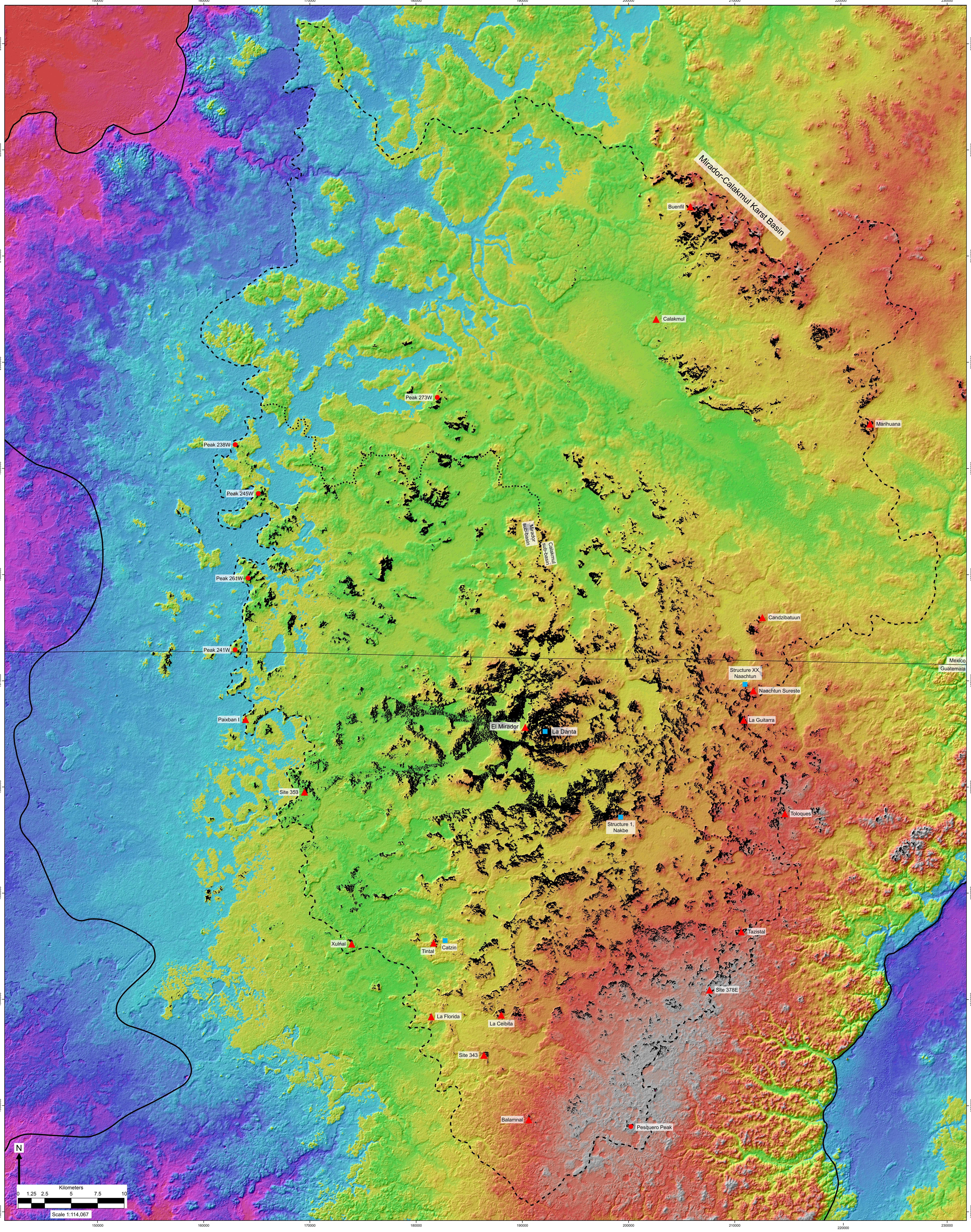

Legend

Elevation (m)
430
110

Petén Plateau
Karst basin
Karst sub-basin
Visible settlement
Visible peak
Maya pyramid

Map Projection: UTM Zone 16N
Datum: WGS 1984
Spheroid: WGS84

ALOS Global Digital Surface Model (AW3D) digital elevation data were provided by the Japan Aerospace Exploration Agency

3D visualization of LiDAR data showing prominent Maya structures created in QGIS.

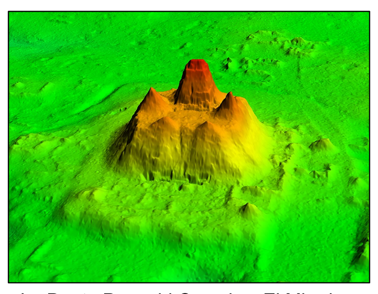
La Danta Pyramid Complex, El Mirador

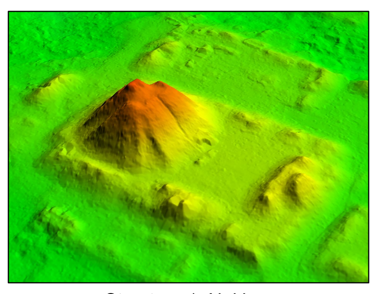
Structure 1, Nakbe

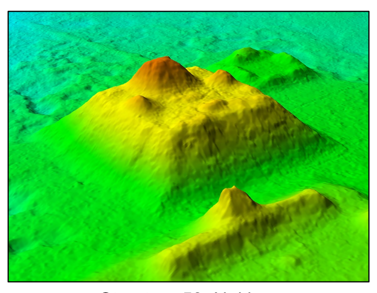
Structure 59, Nakbe

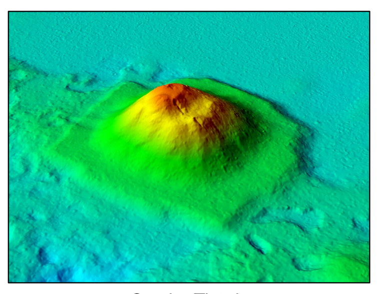
Catzin, Tintal

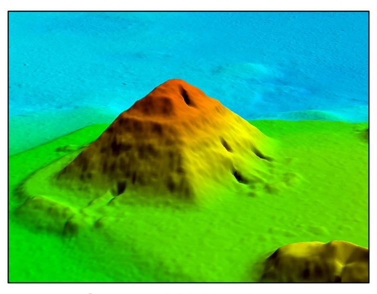
Structure XX, Naachtun

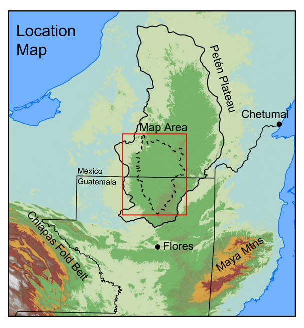

Supplement: S3 Map — AW3D30 elevation data have been provided by JAXA (https://www.eorc.jaxa.jp/ALOS/en/aw3d30/) and printed under a CC BY 4.0 license. LiDAR data have been provided by FARES (https://www.fares-foundation.org/) and printed under a CC BY 4.0 license. All other layers were produced by the authors and are copyright-free. (PDF) [file pone.0255496.s003.pdf]
